# Antipsychotic-induced bone loss: the role of dopamine, serotonin and adrenergic receptor signalling

**DOI:** 10.3389/fcell.2023.1184550

**Published:** 2023-05-25

**Authors:** D. Kavindi Weerasinghe, Jason M. Hodge, Julie A. Pasco, Rasika M. Samarasinghe, Behnaz Azimi Manavi, Lana J. Williams

**Affiliations:** ^1^ IMPACT—The Institute for Mental and Physical Health and Clinical Translation, Deakin University, Geelong, VIC, Australia; ^2^ Barwon Health, Geelong, VIC, Australia; ^3^ Department of Medicine—Western Health, The University of Melbourne, Melbourne, VIC, Australia

**Keywords:** schizophrenia, antipsychotics, osteoclast, osteoblast, bone mineral density, dopamine, serotonin, noradrenaline

## Abstract

Antipsychotics are commonly used in treating psychiatric disorders. These medications primarily target dopamine the serotonin receptors, they have some affinity to adrenergic, histamine, glutamate and muscarinic receptors. There is clinical evidence that antipsychotic use decreases BMD and increases fracture risk, with dopamine, serotonin and adrenergic receptor-signalling becoming an increasing area of focus where the presence of these receptors in osteoclasts and osteoblasts have been demonstrated. Osteoclasts and osteoblasts are the most important cells in the bone remodelling and the bone regeneration process where the activity of these cells determine the bone resorption and formation process in order to maintain healthy bone. However, an imbalance in osteoclast and osteoblast activity can lead to decreased BMD and increased fracture risk, which is also believed to be exacerbated by antipsychotics use. Therefore, the aim of this review is to provide an overview of the mechanisms of action of first, second and third generation antipsychotics and the expression profiles of dopamine, serotonin and adrenergic receptors during osteoclastogenesis and osteoblastogenesis.

## 1 Introduction

**FIGURE 1:**
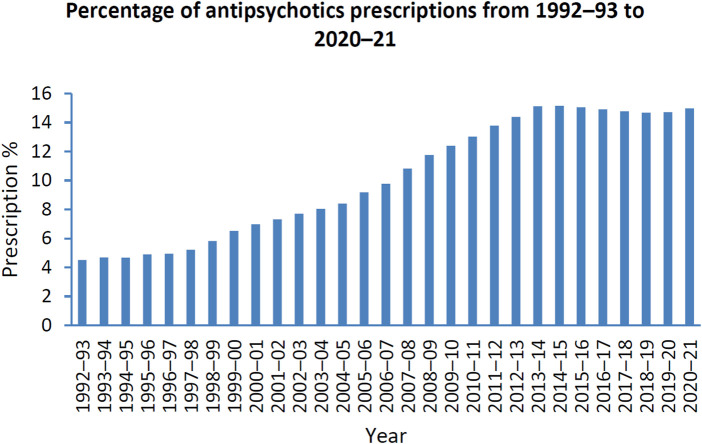
Percentage of patients prescribed antipsychotics from 1992–1993 to 2020–2021; Data adopted from mental health services in Australia: Mental health related prescriptions Australian Institute of Health and Welfare (Australian Institute of Health and Welfare, 2022).

Antipsychotics are the first line treatment in schizophrenia (Lally and MacCabe, 2015). Schizophrenia is a state whereby patients lose connection with reality. This can be in the form of hallucinations, delusions and disorder of thoughts with far-reaching effects on individuals, their families and society (Howes et al., 2009; Patel et al., 2014). Global statistics has shown that schizophrenia is the eighth leading cause of disability in people aged 15–44 years (Haddad and Correll, 2018). Antipsychotics are also used off-label in the treatment of anxiety, borderline personality disorder, substance use disorders, eating disorders, post-traumatic stress disorder, obsessive-compulsive disorder, mood disorders, insomnia, agitation and attention deficit hyperactivity disorder (Wang et al., 2021a; Azimi Manavi et al., 2023). Between 2005–2014, the prescription rate of antipsychotic use overall has increased in 10 out of 16 countries, with the atypical antipsychotics, quetiapine, olanzapine and risperidone being most prescribed (Hálfdánarson et al., 2017). The Australian Institute of Health and Welfare has reported that 17.7% of Australians received mental health-related medications between 2020 and 2021 where the highest prescription rates were 8.9% and 8.7% for antidepressants and antipsychotics, respectively (Australian Institute of Health and Welfare, 2022). Moreover, the prescription of antipsychotics has increased by 10.5% from 1992-93 to 2020-21 (Australian Institute of Health and Welfare, 2022) (Figure 1).

The use of antipsychotics is associated with a number of side effects including tardive dyskinesia (repetitive, involuntary movements commonly in areas of the face, eyes, and mouth), dystonia (involuntary muscle movements that can affect the head, face, and neck), akathisia (inability to remain still), weight gain, hyperglycaemia, type 2 diabetes and adverse cardiovascular impacts including sudden cardiac death (De Hert et al., 2011). There is a growing body of evidence suggesting that antipsychotics are also associated with negative, off-target side effects that include reductions in bone mineral density (BMD) and increased fracture risk (Kishimoto et al., 2008; Crews and Howes, 2012; Fraser et al., 2015; Bolton et al., 2017; May et al., 2019; Paschou et al., 2019; Azimi Manavi et al., 2023). The mechanism(s) by which antipsychotics induce bone loss are yet to be fully explored and understood. This has led to an emerging area of research to understand the underlying mechanisms by which antipsychotics negatively impact bone.

Bone is a multicellular organ consisting of different cell types, namely,: osteoclasts, osteoblasts, osteocytes, chondrocytes, bone lining cells, and bone-resident macrophages known as osteal macrophages or osteomacs (Florencio-Silva et al., 2015; Miron and Bosshardt, 2016; Kenkre and Bassett, 2018; Mohamad et al., 2021). Old and damaged bone is resorbed by osteoclasts while new bone is formed by osteoblasts, which, when entombed in newly formed bone become osteocytes, the most prevalent cells in bone tissue (∼90–95%) that act as a master regulator of osteoclast and osteoblast function (Metzger and Narayanan, 2019). Chondrocytes form the cartilage matrix which is later replaced by osteoblasts to undergo endochondral ossification (Chen et al., 2021), while resting osteoblasts are known as bone lining cells (Lee et al., 2021). Osteomacs also regulate bone modelling and remodelling by acting as potential osteoclast precursors and secreting oncostatin M to promote bone formation by osteoblasts (Walker et al., 2010; Mohamad et al., 2021; Hioki et al., 2022). The fine balance between osteoclast and osteoblast activity is central to maintaining normal bone homeostasis and resultant healthy bone (Florencio-Silva et al., 2015).

Antipsychotics target different receptors including dopamine, serotonin and adrenergic receptors, which are predominantly located in the brain, but have recently been shown to be present in osteoclasts and osteoblasts (Aringhieri et al., 2018). Therefore, this review summarises commonly prescribed antipsychotics and details their mechanisms of action, in addition to how they may influence various dopamine, serotonin and adrenergic receptors shown to be expressed in osteoclasts and osteoblasts that may ultimately lead to bone loss.

## 2 Antipsychotics

Initially, antipsychotics categorised as either “typical” or “atypical.” This has changed first-generation antipsychotics (FGA), second-generation antipsychotics (SGA) and third-generation antipsychotics (TGA). These categories are primarily based on their affinity to dopamine and serotonin receptors, where they act as either agonist, partial agonist, antagonist or an inverse agonist (Strange, 2008). First generation antipsychotics are dopamine D_2_ (D_2_) antagonists, which have been effective in the treatment of positive symptoms while less effective in the treatment of negative symptoms (Mailman and Murthy, 2010; Li et al., 2016). Positive symptoms include hallucinations, delusions and disorder of thoughts while the negative symptoms cause dysfunction in personal, social and occupational functioning through means such as anhedonia, avolition and social withdrawal, as well as, cognitive dysfunction (Winship et al., 2019). The first generation antipsychotic “chlorpromazine” was the first to be recognised as a psychotropic drug in 1952 by Henri Laborit (Laborit et al., 1952; Ban, 2007). Following chlorpromazine, other FGAs including haloperidol, trifluperazine, thioridazine and fluphenazine were introduced (Ramachandraiah et al., 2009). First generation antipsychotics are associated with a number of undesirable side effects including hyperprolactinemia, sedation, weight gain, autonomic and cardiovascular effects, akathisia, parkinsonism, dystonia and tardive dyskinesia (Mailman and Murthy, 2010), thus, spurring on the development of second generation antipsychotics. Second generation antipsychotics are D_2_ and serotonin (5HT) antagonists with affinity ratio greater than 1.12 for D_2_/5HT_2A_ which have been more effective in reducing negative and cognitive symptoms compared to FGAs, but associated with greater weight gain and metabolic syndromes such as hypertriglyceridemia, elevated glucose, insulin and low-density lipoprotein cholesterol levels (Lieberman, 2004; Mailman and Murthy, 2010; Pillinger et al., 2020; Kiss et al., 2022). A third generation of antipsychotics such as aripiprazole, cariprazine and brexpiprazole were introduced most recently and have been successful in treating positive, negative and cognitive symptoms (Vasiliu, 2022). These predominantly act as pre-synaptic and post-synaptic D_2_ partial agonists rather than receptor antagonists, as well as D_3_ and 5HT_1A_ partial agonists, while simultaneously acting as a serotonin 5HT_2A_ and 5HT_2B_ antagonist (Li et al., 2016; Kiss et al., 2022). Current research is focusing on D_3_ targeting antipsychotics due to their higher affinity for dopamine than D_2_ receptors (Maramai et al., 2016). Moreover, D_3_ are predominantly expressed in the limbic system, which is involved in schizophrenia pathology (Kiss et al., 2022) and the blockage of the D_3_ is associated with pro-cognitive effects in rats (Watson et al., 2012). As shown in Table 1, antipsychotics possessaffinity affinity to other receptors including adrenergic, histamine and muscarinic receptors (Table 1).

**TABLE 1:** Receptor binding profiles for commonly prescribed antipsychotics.

	Antipsychotics	Therapeutic plasma level	D_1_	D_2_	D_3_	D_4_	5HT_1A_	5HT_1B_	5HT_2A_	5HT_2B_	5HT_2C_	5HT_6_	5HT_7_	Adra1	Adra2a	Adra2b	Adra2c	Adrb2	H1	H2	H3	M1	M3
(A) Receptor binding profiles for commonly prescribed first generation antipsychotics																							
FGA	Chlorpromazine	23–600 ng/mL Chetty and Miller. (1991)	**++**	**+++**	**+++**	**+++**			**+++**	**+++**	**++**	**+++**	**+++**	**+++**	**+**	**++**	**++**	**?**	**+++**	**+**	**+**	**++**	**++**
	Haloperidol	2–12 ng/mL Van Putten et al. (199). Above 18 ng/mL counter therapeutic Coryell et al. (1998)	**+**	**+++**	**+++**	**+++**		**+**	**+**				**+**	**++**	**+**	**+**	**+**			**+**			
	Periciazine	1–5 nmol/L Omérov et al. (1989)	**+**	**++++**			**+**		**+++**		**+**			**++**	**+**				**+++**				
	Loxapine	—	**++**	**+++**	**++**	**++**		**+**	**+++**		**++**	**++**	**++**										
	Zuclopenthixol	—		**+++**															**+++**				
	Flupentixol	—																					
	Trifluoperazine	0.53–3.09 ng/mL Midha et al. (1983)		**+++**	**++++**	**++**	**+**		**++**		**+**	**+**	**+**										
	Perphenazine	1–5 nmol/L Omérov et al. (1989)	**?**	**++++**			**+**		**+++**		**+**	**++**	**++**										
	Thiothixene	—	**++**	**+++**	**+**	**+++**	**+**	**+**	**++**			**+**	**++**										

	Antipsychotics	Therapeutic plasma level	D_1_	D_2_	D_3_	D_4_	5HT_1A_	5HT_1B_	5HT_2A_	5HT_2B_	5HT_2C_	5HT_6_	5HT_7_	Adra1	Adra2a	Adra2b	Adra2c	Adrb2	H1	H2	H3	M1	M3
(B) Receptor binding profiles for commonly prescribed second generation antipsychotics																							
SGA	Clozapine	350–420 ng/mL Papola et al. (2018)	**+**	**+**	**+**	**++**	**+**	**+**	**++**	**+++**	**++**	**++**	**++**	**+++**	**++**	**++**	**++**		**+++**	**+**		**+++**	**++**
	Olanzapine	20–80 ng/mL May et al. (2019)	**++**	**++**	**++**	**++**		**+**	**+++**	**++**	**++**	**+++**	**++**	**++**	**+**	**++**	**++**		**+++**	**++**	**+**	**++**	**++**
	Quetiapine	100–500 ng/mL Houseknecht et al. (2017)	**+**	**+**	**+**		**++**		**++**		**+**	**+**	**++**	**++**	**+**	**+**	**++**		**+++**			**+**	**+**
	Amisulpride	100–319 ng/mL Kawamura et al. (2011)		**+++**	**+++**	**+++**				**++**			**++**										
	Risperidone	20–60 ng/mL Papola et al. (2018)	**+**	**+++**	**+++**	**+++**	**+**	**++**	**++++**	**++**	**++**		**+++**	**+++**	**++**	**++**	**+++**		**+++**	**+**			
	Lurasidone	No data available	**+**	**+++**	**?**		**+++**		**+++**		**+**		**++++**	**++**	**++**		**+++**		**?**				
	Ziprasidone	50–130 ng/mL Stubbs et al. (2015)	**++**	**+++**	**++**	**+**	**++**	**+++**	**++++**		**++**	**++**	**+++**	**+++**					**+++**			**+**	
	Paliperidone	20–60 ng/mL Cohen et al. (2019)	**+**	**+++**			**+**		**+++**		**++**			**+++**					**+++**			**+**	
	Iloperidone	>4 ng/mL provide clinical efficacy ClinicalTrials.gov. (2014)	**+**	**+++**	**+++**	**++**	**+**		**+++**		**++**	**++**	**+**	**+++**			**++**		**+**				
	Asenapine	—	**+++**	**+++**	**++++**	**+++**	**+++**	**+++**	**++++**		**++++**	**++++**	**++++**	**+++**					**+++**				

	Antipsychotics	Therapeutic plasma level	TAAR 1	D_1_	D_2_	D_3_	D_4_	5HT_1A_	5HT_1B_	5HT_2A_	5HT_2B_	5HT_2C_	5HT_6_	5HT_7_	Adra1	Adra2a	Adra2b	Adra2c	Adrb2	H1	H2	H3	M1	M3
(C) Receptor binding profiles for commonly prescribed third generation and other antipsychotics																								
TGA	Aripiprazole	150–300 ng/mL Lahdelma et al. (2010)			**++++**	**++++**	**++**	**+++**	**+**	**+++**	**++++**	**++**		**++**	**++**	**++**	**++**	**++**		**++**				
	Cariprazine	—			**++++**	**++++**		**+++**	**?**	**++**	**++++**	**+**	**?**	**+**	**+**					**++**			**?**	
	Brexpiprazole	—			**++++**	**+++**		**++++**		**++++**	**+++**	**++**		**+++**	**+++**	**?**	**?**	**++++**		**++**			**+**	
Other	Ropinirole	0.4–6.0 ng/mL Motyl et al. (2012); Li et al. (2019)			**+++**	**+++**	**+++**																	
	Ulotaront	Undergoing clinical trials	**?**					**?**																

Antagonism and inverse agonism are indicated by blue, agonism by green and partial agonism by yellow. Data taken from (34,64,140–142,147–184). Binding affinity for receptors: + = week association (Ki value 100–1,000); ++ = Moderate association (10–91); +++ = strong association (Howes et al., 2009; De Hert et al., 2011; Patel et al., 2014; Fraser et al., 2015; Lally and MacCabe, 2015; Hálfdánarson et al., 2017; Haddad and Correll, 2018; Wang et al., 2021a; Australian Institute of Health and Welfare, 2022; Azimi Manavi et al., 2023); ++++ = very strong association (Ki < 1); ? = unknown.

TAAR1 = Trace amine associated receptor 1, D = Dopamine receptor, 5HT = Serotonin receptor, Adr = Adrenergic receptor, H= Histamine receptor, M = Muscarinic receptor.

## 3 Schizophrenia and bone health: epidemiological findings

There is a growing body of evidence to show that schizophrenia is associated with osteoporosis, a degenerative bone disease characterised by low BMD and increased fracture risk (Jung et al., 2011; Lally, 2020; Forns et al., 2021; Azimi Manavi et al., 2023). The onset of schizophrenia occurs primarily in adolescents and young adults, where peak bone mass is yet to be achieved (Forns et al., 2021; Zhu and Zheng, 2021). Patients with schizophrenia are possibly vulnerable to bone defects due to lifestyle factors such as a reduced physical activity, smoking, alcohol consumption, vitamin D deficiency and poor dietary habits in addition to genetic and biological factors (Coentre et al., 2009). A study has shown that smoking and alcohol consumption also leads to further reduced BMD in patients with schizophrenia compared to those who do not smoke or drink alcohol (Jung et al., 2011). Moreover, studies show that the use of antipsychotics further exacerbates bone loss and fracture risk in patients with schizophrenia (Oderda et al., 2012; Rigler et al., 2013; Fraser et al., 2015; Bolton et al., 2017; May et al., 2019; Paschou et al., 2019; Shen et al., 2019). Similarly, studies have shown that increased bone fractures are linked with schizophrenia compared to controls (Tsai et al., 2014; Stubbs et al., 2015; Tseng et al., 2015).

## 4 Antipsychotic use and bone health: epidemiological, *in vitro* and *in vivo* findings

Epidemiological evidence is growing to suggest antipsychotic use (independent of schizophrenia) is associated with lower BMD, increased fracture risk (Oderda et al., 2012; Rigler et al., 2013; Fraser et al., 2015; Bolton et al., 2017; May et al., 2019; Paschou et al., 2019; Shen et al., 2019; Azimi Manavi et al., 2023) and occurrence of re-fracture (Shen et al., 2019). Some studies suggest typical antipsychotics have a higher fracture risk compared to atypical antipsychotics (Ching-Min et al., 2020), while others suggest there is no difference with regards to the type of antipsychotics and associated fracture risk (May et al., 2019; Shen et al., 2019). Risperidone has been shown to reduce BMD in children and young adults (Calarge et al., 2010; Rosenbloom, 2010) with a significant decrease in BMD compared to olanzapine or other SGAs (Becker et al., 2003; Clapham et al., 2020). It has been reported that patients receiving either FGAs or SGAs are at higher fracture risk as antipsychotics in these categories such as chlorpromazine, risperidone and zotepine increased incident fracture, as well as, re-fracture (Ching-Min et al., 2020). A meta study concluded that FGA users show a 77% increase in hip fractures compared to non-users, while SGA users show a 41% increase. This study also noted that associated fracture risk is 29%, 46%, 49% and 94% for risperidone, olanzapine, quetiapine and haloperidol, respectively (Papola et al., 2018; Shen et al., 2019). Interestingly, research has shown that antipsychotics can accumulate up to 15-fold higher concentration in bone marrow compared to plasma (Houseknecht et al., 2017; May et al., 2019).

Evidence from *in-vitro* (Lahdelma et al., 2010; Kawamura et al., 2011; Motyl et al., 2012; Li et al., 2019) and *in-vivo* bone models (Oh-ie et al., 2002; Kunimatsu et al., 2010; Kawamura et al., 2011; Motyl et al., 2012) support these findings. In experiments conducted in rat primary cells, clozapine inhibited osteoblast mitogenesis and differentiation, as well as, osteoclast formation, whereas haloperidol had no effect (Costa et al., 2011). In mouse bone marrow mesenchymal stem cells (BMSCs), chlorpromazine inhibited osteoclastogenesis via inhibiting the intracellular calmodulin pathway (Kawamura et al., 2011). Risperidone significantly stimulated osteoclast differentiation without affecting osteoblast differentiation (Motyl et al., 2012). *In vitro* studies on antipsychotic-related bone defects in primary human cells are scant. A study showed clozapine has cytotoxic effects on primary cultures of human BMSCs (Lahdelma et al., 2010). A study using the human osteoblast cell line hFob1.19 showed that risperidone, amisulpride, olanzapine and aripiprazole inhibited osteoblasts cell viability and caused apoptosis in a dose dependent manner (Li et al., 2019).

*In vivo* evidence has similarly shown negative effects of antipsychotics on bone. Risperidone reduced trabecular and cortical bone mass in mice with increased osteoclast differentiation and resorption using bone marrow derived cells (Motyl et al., 2012). Chlorpromazine and haloperidol have also been shown to decrease BMD in trabecular bone of mice via increased resorption (Oh-ie et al., 2002; Kunimatsu et al., 2010). However, this induced bone loss through dopamine blockers is considered to be rodent specific (Kunimatsu et al., 2010). Clozapine, but not haloperidol, decreased BMD *in-vivo* in mice and reduced osteoclast and osteoblast activity *in-vitro* (Kawamura et al., 2011). Chlorpromazine also reduce osteoclast formation and osteoclastic gene expression in mouse BMSCs (Lahdelma et al., 2010).

## 5 Antipsychotics and bone cell function: importance of dopamine receptors/signalling in osteoclasts and osteoblasts

Dopamine is a neurotransmitter that controls sleep, motivation, reward, attention, voluntary movements, vision, hormonal regulation and motor functions (Hornykiewicz, 2017; Yang and Tsai, 2017; Wang et al., 2020). Abnormalities in the dopamine system are associated with schizophrenia and psychosis, where positive symptoms are linked with increased dopamine levels in the mesolimbic pathway associated with dopamine D_2_ hyper-activity (McCutcheon et al., 2018; Stahl, 2018; McCutcheon et al., 2019). Accordingly, most antipsychotics have been developed to target dopamine D_2_. Decreased levels of dopamine in cortical pathway has also been observed using PET scanning in patients with schizophrenia, which needs further study as it is believed that hypo-activity of the mesocortical pathway is associated with negative and cognitive symptoms in schizophrenia (Li et al., 2016; Weinstein et al., 2017). Even though this theory has been accepted for decades, recent data indicate the involvement of dorsal striatum in pathophysiology of schizophrenia (McCutcheon et al., 2019).

**FIGURE 2:**
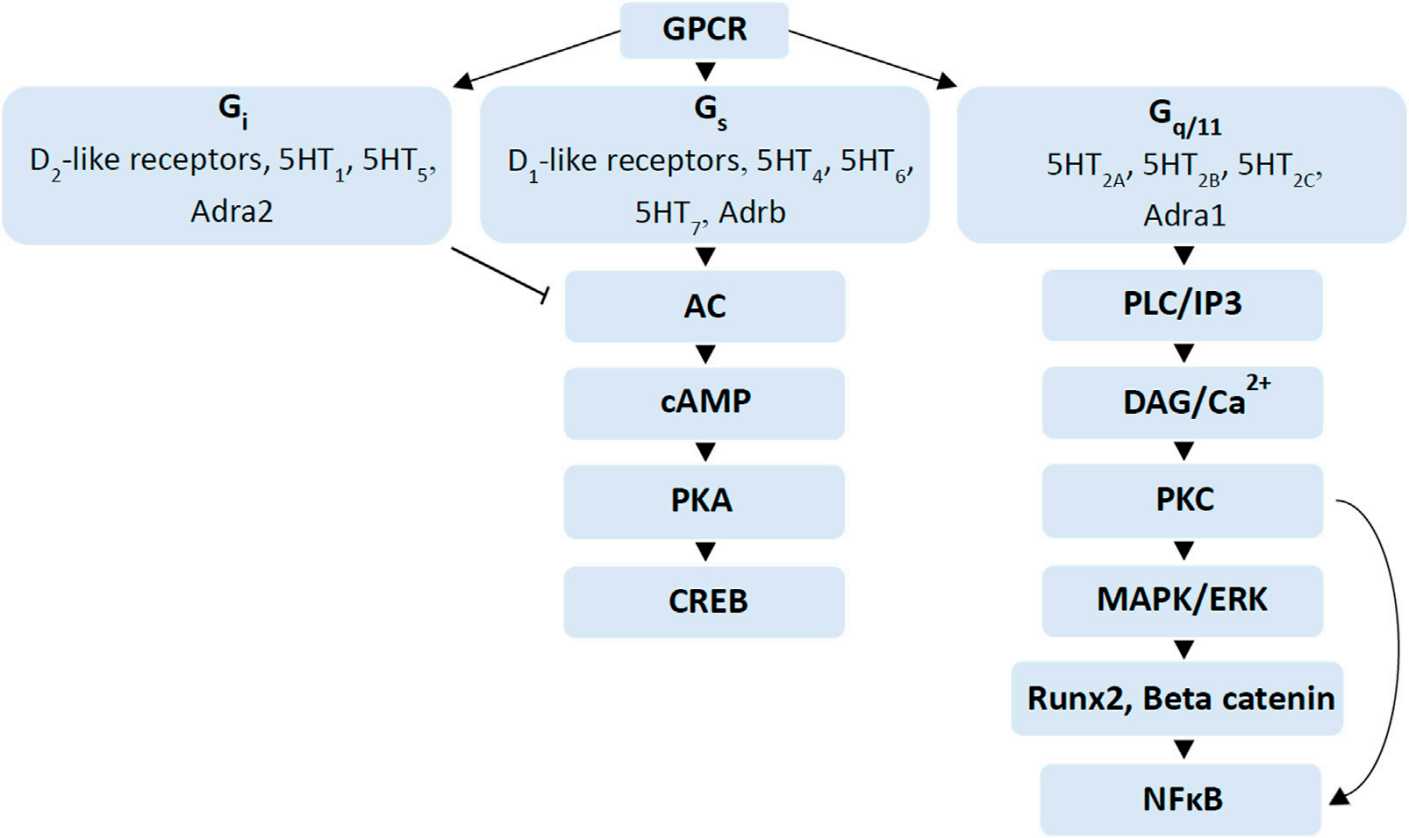
Signalling cascades regulated by dopamine, serotonin and adrenergic receptors. G-protein coupled receptor stimulation by different neurotransmitters such as dopamine, serotonin and adrenergic receptors regulate different signalling cascades. Activation of G_i_ receptors such as D_2_-like receptors, 5HT_1,_ 5HT_5_ and Adra2 inhibit adenylate cyclase (AC), while activation of G_s_ receptors such as D_1_-like receptors, 5HT_4_, 5HT_6_, 5HT_7_ and Adrb stimulate AC to increase cAMP levels, which in turn activate the PKA/CREB pathway (Zhao et al., 2016; Ramaswamy et al., 2017; Wang et al., 2018; Martel and Gatti McArthur, 2020). G_q/11_ receptors activate PLC-IP3 and DAG-PKC signalling pathways, which activate the mitogen-activated protein kinase (MAPK)/ERK signalling cascade (Gustafsson et al., 2006a; Dai et al., 2014; McCorvy and Roth, 2015). Activation of MAPK/ERK also stimulates Runx2 and beta-catenin in osteogenesis while activation of Protein kinase C (PKC) stimulates nuclear factor kappa-light-chain-enhancer of activated B cells (NFκB), important in osteoclast differentiation (Abu-Amer, 2013; Zhao et al., 2016; Kim et al., 2019).

Dopamine receptors are G-protein-coupled receptors (GPCR), which belongs to GPCR alpha subunits G_s_ (stimulatory receptors) and G_i_ (inhibitory receptors) (Zhang and Shi, 2016; Martel and Gatti McArthur, 2020). There are five dopamine receptors, which are sub-categorised as D_1_-and D_2_-like, depending on pharmacology and ability to regulate cyclic adenosine monophosphate (cAMP) (Martel and Gatti McArthur, 2020). Dopamine D_1_ and D_5_ belong to D_1_-like receptors that modulate signalling pathways via G_s_ and increase cAMP production while D_2_, D_3_ and D_4_ belong to D_2_-like receptors that modulate via G_i_ and inhibit cAMP production (Figure 2) (Neve et al., 2004; Martel and Gatti McArthur, 2020). Moreover, D_2_-like receptors have 10- to 100-fold higher affinity for dopamine than D_1_-like receptors (Martel and Gatti McArthur, 2020). This leads to the assumption that extracellular dopamine levels or changes to dopamine levels from antipsychotic use may have different impacts on D_2_-like receptors compared to D_1_-like receptor signalling (Martel and Gatti McArthur, 2020). Dopamine has been found in bone marrow in higher concentrations (∼10 µM) than typically found in circulation (Matt and Gaskill, 2020). Moreover, hypothalamic neurons produce dopamine to regulate bone metabolism via hypothalamic-pituitary-gonadal axis while sympathetic nerves also contain dopamine which can penetrate to bone marrow (Wang et al., 2021b).

### 5.1 Dopamine signalling in osteoclasts

Dopamine has been demonstrated to inhibit osteoclastogenesis in a dose-dependent manner using osteoclast precursors derived from: i) human CD14^+^ cells isolated from peripheral blood mononuclear cells (PBMCs) (Hanami et al., 2013); ii) murine BMSCs (Yang et al., 2016); and iii) RAW 264.7 cells (Wang et al., 2021b). There are limited studies showing links between dopamine and bone cell function. Studies have reported that dopamine can interact with receptors in osteoclasts derived from human BMSCs to inhibit resorption via nuclear factor of activated T-cells cytoplasmic 1 (NFATc-1) and c-Fos signalling pathways (Hanami et al., 2013; Wang et al., 2020). Hanami et al. (2013) showed that dopamine D_2_-like signalling inhibits osteoclastogenesis *in-vitro*, which could be a protective mechanism against bone resorption while Wang et al. (2021) showed that dopamine inhibits osteoclastogenesis via D_2_R/cAMP/protein kinase A (PKA)/cAMP response element-binding protein (CREB) pathway in primary mice BMSCs (Wang et al., 2021b). In contrast, it has been shown that D_1_-like receptor antagonist SCH23390 inhibits osteoclastogenesis in mice bone marrow-derived macrophages (Nakashioya et al., 2011).

Expression of dopamine receptors in osteoclast has been reported in a number of animal and human models (Table 2). The presence of all five dopamine receptors and the dopamine transporter (DAT) have been reported in human CD14^+^ monocytes at both transcriptional and protein levels (Gaskill et al., 2012; Hanami et al., 2013). Maximum expression was observed in the early stages of osteoclastogenesis, decreasing after day 7 (Hanami et al., 2013). Similarly, all five dopamine receptors were identified in RAW 264.7 cells and in primary mice bone marrow derived macrophage-lineage cells from C57BL/6J mice (Wang et al., 2021b), in addition to mouse BMSCs, where increased expression of D_1_, D_2_ and D_3_ was observed during osteoclastogenesis (Cheong et al., 2018).

**TABLE 2:** Dopamine receptor expression in osteoclasts and osteoblasts.

Type of dopamine receptor	Experiment cell type	References
Osteoclasts (OC)		
D_1_, D_2_, D_3_, D_4_ and D_5_	RAW 264.7 cells	Wang et al. (2021b)
D_1_, D_2_, D_3_, D_4_ and D_5_	Primary mice BM cells	Berger et al. (2009), Motyl et al. (2017), Cheong et al. (2018), Wang et al. (2021b)
D_1_, D_2_, D_3_, D_4_, D_5_ and DAT	Human PBMC derived CD14^+^ cells	Gaskill et al. (2012), Hanami et al. (2013), Wysokiński et al. (2021)
Osteoblasts (OB)		
D_1_, D_2_, D_3_, D_4_ and D_5_	MC3T3-E1 cells	Lee et al. (2015), Motyl et al. (2017), Zhu et al. (2022)
D_1_ and D_2_	Rat BMSCs	Cheong et al. (2018), Wang et al. (2020)
D_1_, D_2_, D_3,_ D_4_ and D_5_	Mice primary BM derived OB	Motyl et al. (2017), Handa et al. (2019)
D_1_ and D_2_	Human BMSCs	Wang et al. (2020)

### 5.2 Dopamine signalling in osteoblasts

The effects of dopamine on osteoblasts is more contentious. A study by Lee et al. (2015) reported that dopamine enhances osteoblast proliferation and mineralisation *in-vitro* in a MC3T3-E1 pre-osteoblastic murine cell line (Lee et al., 2015), while another study by Motyl et al. (2017) directly refuted these findings, reporting suppression of osteoblast mineralisation and osteoblast markers of gene expression (Motyl et al., 2017). The expression of all five dopamine receptors in osteoblasts has been shown at both mRNA and protein levels in MC3T3-E1 cells (Lee et al., 2015; Zhu et al., 2022). Dopamine (50 µM) did not affect osteoblast cell proliferation but increased mineralisation, compared to control, whereas concentrations above 100 µM induced cell death (Lee et al., 2015). Bone marrow mesenchymal stem cells can be differentiated into cartilage, tendon, muscle, fat and osteoblasts (Caplan, 1991; Pittenger et al., 1999; Sacchetti et al., 2007; Méndez-Ferrer et al., 2010; Wang et al., 2020). Primary murine BMSCs-derived osteoblasts express dopamine receptors, which regulate proliferation and differentiation (Motyl et al., 2017; Cheong et al., 2018; Handa et al., 2019; Wang et al., 2020). The expression of D_1_ and D_2_ was significantly increased from day 3 to day 7 in rat BMSCs (Wang et al., 2020). Wang et al. (2020) showed the presence of dopamine receptors D_1_ and D_2_ in human BMSCs where the expression of D_1_ increased until day 5 and the expression of D_2_ decreased from day three to day 5 (Wang et al., 2020). Moreover, alkaline phosphatase activity (ALP), mineralisation and expression of bone sialoprotein, runt-related transcription factor 2 (Runx2), and osteocalcin were stimulated with dopamine (5 nM). This stimulation was lost at 50 µM, whereas 500 μM was toxic (Wang et al., 2020). This study also demonstrated that dopamine-induced osteogenic differentiation of human BMSCs was mediated through D_1_ receptor activation via extracellular signal-regulated kinase (ERK) 1 and 2 signalling pathways and activation of Runx2 transcriptional activity, while the suppression of cAMP/PKA pathway may inhibit dopamine-induced human BMSCs differentiation (Wang et al., 2020).

Antipsychotics targeting D_3_ receptors have gained more attention in treating impaired cognitive, social, and motor functions associated with schizophrenia. To our knowledge, there are no studies on the effects of D_3_ on bone remodelling. Hence, it is important to investigate the effects of D_3_ in bone cells function, due to its similarity to D_2_, which can also activate ERK/MAPK signalling pathway (Kiss et al., 2022), which is important in bone homeostasis and skeleton development (Kim et al., 2019). Moreover, both D_2_ and D_3_ activate their signalling cascades in GPCR independent manner via beta arresting mediated pathways (van Gastel et al., 2018). Studies show that beta arrestin 2 is involved in regulating MAPK/AKT/glycogen synthase kinase 3 (GSK3) pathways downstream of the dopamine receptors (Del'Guidice et al., 2011). Glycogen synthase kinase 3 is expressed in the brain and important in psychiatric diseases such as schizophrenia (van Gastel et al., 2018). Interestingly, both D_2_ and D_3_ are positively coupled to beta arrestin/AKT/GSK3 pathway (van Gastel et al., 2018). Beta arrestin 2 stimulates mice trabecular bone formation but not bone resorption indicating the importance of this pathway in bone homeostasis (Gesty-Palmer et al., 2009).

In summary, the presence of all dopamine receptors in osteoclasts has been shown using RAW 264.7, primary mice BMSCs and human CD14^+^ cells where expression increased during osteoclastogenesis (Table 1). The presence of dopamine receptors in osteoblasts has also been shown in MC3T3-E1 (with no difference in expression level for 14 days), rat BMSCs (with increased D_1_ and D_2_ from day 3 to 7) and human BMSCs (with increased D_1_ expression until day 5 while D_2_ expression decreased after day 3) (Lee et al., 2015; Wang et al., 2020). It has also been demonstrated that low concentrations of dopamine (5 nM) can increase osteoblast marker expression (Wang et al., 2020). Moreover, dopamine regulates osteoclastogenesis via nuclear factor of activated T Cells 1 (NFATc1), cFOS, cAMP/PKA/CREB via D_2_ receptor while dopamine regulates osteoblastogenesis via ERK 1 and 2, Runx2 and cAMP/PKA via the D_1_ receptor (Wang et al., 2020; Wang et al., 2021b). However, there is limited data available on the effect of dopamine on human models. As all antipsychotics primarily target dopamine receptors as antagonists or partial agonists it is important to understand the dopamine receptor expression profile in bone cells to understand the complex mechanism of antipsychotics-induced bone loss.

## 6 Antipsychotics and bone cell function: importance of serotonin receptors/signalling in osteoclasts and osteoblasts

Serotonin is a product of tryptophan metabolism and is produced both in the central nervous system and peripherally in gastrointestinal tract and in platelets, where it does not cross the blood brain barrier (Brenner et al., 2007; Kanova and Kohout, 2021). Peripheral serotonin accounts for the majority of serotonin produced in the body (∼95%), while the brain is responsible for only a minor fraction (Ducy and Karsenty, 2010; El-Merahbi et al., 2015). Centrally, serotonin acts as a neurotransmitter, while peripherally its role is as a hormone (Kanova and Kohout, 2021). As a neurotransmitter, serotonin regulates mood, reward, anger, perception, appetite, aggression, attention, memory and sex drive. Peripherally, serotonin regulates major organ functions including glucose homeostasis and lipid metabolism (Berger et al., 2009; Kanova and Kohout, 2021). It has been reported that serotonin acts centrally to inhibit bone resorption and increase bone formation, whilst peripherally it acts to directly inhibit bone formation (Ducy and Karsenty, 2010; El-Merahbi et al., 2015). It has been reported that osteoclasts can synthesise serotonin in the presence of receptor activator of nuclear factor kappa-Β ligand (RANKL). Osteoblast-derived RANKL stimulates the expression of tryptophan hydroxylase 1 (TPH_1_) by osteoclasts, which results in serotonin synthesis that may then play a role as a paracrine/autocrine factor to further regulate osteoclast formation and resorption (Chabbi-Achengli et al., 2012).

There are seven types of serotonin receptors, 5HT_1_ to 5HT_7,_ which are all GPCRs, except 5HT_3_ (Nichols and Nichols, 2008; McCorvy and Roth, 2015). These receptors modulate different signalling pathways via G_i_, guanine nucleotide binding protein alpha 11 (G_q/11_), and G_s_ (Figure 2) (McCorvy and Roth, 2015). The receptors 5HT_1_ and 5HT_5_ belong to the G_i_ protein-coupled receptors, while the 5HT_2A_, 5HT_2B_ and 5HT_2C_ receptors belong to the G_q/11_ protein-coupled receptors and 5HT_4_, 5HT_6_ and 5HT_7_ belong to G_s_ protein-coupled receptors (Dai et al., 2014; McCorvy and Roth, 2015). The G_i_ protein-coupled receptors 5HT_1_ and 5HT_5_ suppress the cAMP/PKA pathways, inhibiting bone formation (Yadav et al., 2008; Dai et al., 2014; McCorvy and Roth, 2015). It has also been shown that activation of the cAMP/PKA pathway is associated with increased osteogenesis *in-vitro* and increased bone formation *in-vivo* (Siddappa et al., 2009). In contrast, in OC, the inhibition of PKA leads to the phosphorylation of activating transcription factor 4 (ATF4), which stimulates osteoclast differentiation (Kode et al., 2012). G_q/11_ protein-coupled receptors, 5HT_2A_, 5HT_2B_ and 5HT_2C_ activate phospholipase C-inositol phosphate 3 (PLC-IP3) and diacylglycerol-protein kinase C (DAG-PKC) signalling pathways, which promote osteoblast differentiation and bone formation while G_s_ protein-coupled receptors 5HT_4_, 5HT_6_ and 5HT_7_ increase cAMP levels (Gustafsson et al., 2006a; Dai et al., 2014; McCorvy and Roth, 2015).

### 6.1 Serotonin signalling in osteoclasts

The localisation of serotonin receptors has been extensively reported in animal and human *in-vitro* and *in-vivo* bone cell models, with varying expression profiles of serotonin receptors in osteoclasts (Table 3). Expression of 5HT_1B_, 5HT_2A_, 5HT_4_ and serotonin transporter (SLC6A4) has been reported in differentiated RAW264.7-derived osteoclasts (Battaglino et al., 2004), while the presence of all serotonin receptors (5HT_1_- 5HT_7_) have been demonstrated in mouse BMSCs, suggesting a role for these receptors in osteoclasts differentiation (Park et al., 2018). The presence of 5HT_1B_, 5HT_2A_, and 5HT_2B_ was shown in osteoclast precursors from mice spleen cells and not 5HT_1A_ and 5HT_4_ (Chabbi-Achengli et al., 2012). The expression level changed with RANKL exposure where 5HT_1B_ receptor expression increased and 5HT_2B_ expression decreased with no change in the 5HT_2A_ receptor expression (Chabbi-Achengli et al., 2012). This could suggest that 5HT_1B_ may be important in osteoclast differentiation from precursors to mature osteoclasts while 5HT_2A_ may act before osteoclasts lineage begins (Chabbi-Achengli et al., 2012). Gustafsson et al. (2006) showed the presence of 5HT_2A_, 5HT_2B_, 5HT_2C_, SLC6A4 and TPH_1_ but not 5HT_1A_ in human PBMC-derived osteoclasts (Gustafsson et al., 2006b). The presence of 5HT_1B_, 5HT_2B,_ SLC6A4 and TPH_1_ in human umbilical cord blood-derived osteoclast precursors have been shown, while the level of 5HT_2B_ expression increased in mature osteoclasts (Hodge et al., 2013).

**TABLE 3:** Serotonin receptor expression in osteoclasts and osteoblasts.

Type of serotonin receptor	Experiment cell type	References
Osteoclasts (OC)		
5HT_1B_, 5HT_2B_, 5HT_4_ and SLC6A4	RAW264.7	Battaglino et al. (2004)
5HT_1B_, 5HT_2A_, and 5HT_2B_, but not 5HT_1A_ and 5HT_4_	Primary mice spleen cell cultures composed mainly of monocytes/macrophages	Chabbi-Achengli et al. (2012)
5HT_1_- 5HT_7_	Mice BM derived macrophages	Park et al. (2018)
5HT_2A_, 5HT_2B_, and 5HT_2C_, SLC6A4, and TPH1, but not 5HT_1A_	Human PBMC	Gustafsson et al. (2006b)
5HT_1B_, 5HT_2B_, SLC6A4 and TPH1, but not 5HT_2A_	Human CBMC	Hodge et al. (2013)
Osteoblasts (OB)		
SLC6A4, 5HT_1A_, 5HT_2A_ and TPH1	MLO-Y4 and MC3T3-E1	Bliziotes et al. (2006)
SLC6A4, 5HT_1A_, 5HT_1D_, 5HT_2A_ and 5HT_2B_	Primary rat OB, ROS 17/2.8, UMR 106-H5, and Py1a	Bliziotes et al. (2001)
5HT_2A_, 5HT_2B_ and 5HT_2C_	Mouse OB from the limbs (tibiae, femurs, humeri) of adult swiss albino mice	Westbroek et al. (2001)
5HT_2B_	Fetal chicken calvarial bones	
5HT_1A_, 5HT_1B_, 5HT_2A_ and 5HT_2B,_ 5HT_6_	Primary mouse OB	Collet et al. (2008), Yadav et al. (2008), Yun et al. (2016)
5HT_1A_, 5HT_1B_, 5HT_1D_, 5HT_2A_, 5HT_2B_ 5HT_2C_	Primary rat OB	Dai et al. (2014)
TPH1, SLC6A4,5HT_1B_, 5HT_2A_ and 5HT_2B_	Primary Human OB from trabecular bone samples	Hodge et al. (2013)

Serotonin has been shown to increase osteoclastogenesis by stimulating both osteoclast formation and resorption in human PBMC derived osteoclasts (Gustafsson et al., 2006b). It has been reported that RANKL induces serotonin synthesis in mice osteoclast precursors whereas serotonin synthesis decreased as osteoclasts matured (Chabbi-Achengli et al., 2012). The study by El-Merahbi, Löffler et al. (2015) also found that 5HT_1B_ and 5HT_2A_ are involved in mediating serotonin in osteoclast differentiation. Moreover, selective blockage of 5HT_1B_, 5HT_2B_ and 5HT_4_ did not affect osteoclastogenesis in RAW264.7 cells (Battaglino et al., 2004). In contrast, antagonism at 5HT_1B_ and 5HT_4_ in mouse BMSCs-derived osteoclasts inhibit osteoclast marker expression (Battaglino et al., 2004). This may be because RAW264.7 cells are more differentiated than osteoclast precursors used in bone marrow cell preparation, also suggesting the importance of 5HT_1B_ and 5HT_4_ in mediating osteoclast differentiation (Battaglino et al., 2004). The osteoclasts activity was inhibited by serotonin or serotonin agonists for 5HT_1_, 5HT_4_, and 5HT_7_, with no effect on osteoclasts activity with 5HT_2_ and 5HT_5_ agonists in mice BMSCs (Park et al., 2018). Serotonin has been shown to increase osteoclastogenesis at concentrations of 10 μM or below in mice BMSCs derived osteoclasts, with 5HT_6_ being more significant (Park et al., 2018). Moreover, it has been reported that mast cells release serotonin locally at high concentration (Battaglino et al., 2004). Mast cells in bone marrow are reported to increase in ovariectomised rats that suffered rapid bone loss (Lesclous et al., 2001). These data suggest that serotonin may modulate osteoclastogenesis *in-vivo*.

### 6.2 Serotonin signalling in osteoblasts

The expression of SLC6A4, 5HT_1A_, 5HT_1B_, 5HT_1D_, 5HT_2A_, 5HT_2B_, 5HT_2C_ and TPH_1_ have been reported in primary cultures of rat osteoblasts, in addition to the osteoblastic cell lines; ROS 17/2.8, UMR 106-H5, Py1a, MLO-Y4 and MC3T3-E1, with the highest expression of 5HT_1B_ and 5HT_2A_ observed in both the early and late stages of differentiation (Bliziotes et al., 2001; Dai et al., 2014). The presence of TPH_1_ indicates that these cell lines may have the capacity to synthesise serotonin that may subsequently act in a paracrine/autocrine manner on bone cells in the bone remodelling space. Receptor 5HT_2B_ has been demonstrated in fetal chicken bone, where this receptor had the highest mRNA expression in skeleton tissue compared to the other tested tissues (Westbroek et al., 2001). The presence of 5HT_2A_, 5HT_2B_ and 5HT_2C_ also observed in murine osteoblasts, where 5HT_2A_ and 5HT_2B_ had higher mRNA expression compared to 5HT_2C_ (Westbroek et al., 2001). Serotonin decreased osteoblast proliferation and mineralisation from 1 nM to 10 μM, where there is an increased trend of proliferation and mineralisation after 1 µM (Dai et al., 2014). Collet et al. (2008) reported 5HT_2B_ receptor expression increased during osteoblast differentiation in bone marrow derived mouse osteoblasts while 5HT_2B_^−/-^ leads to osteopenic phenotype (Collet et al., 2008). Yadav et al. (2008) also reported the presence of 5HT_1B_, 5HT_2A_ and 5HT_2B_ receptors in primary mouse osteoblasts and that serotonin inhibited osteoblast proliferation via 5HT_1B_ by inhibiting cAMP/PKA/CREB while there was no effect of 5HT_2A_ and 5HT_2B_ on bone mass (Yadav et al., 2008). In contrast, it has been shown that 5HT_2B_ receptor activation was responsible for increased ALP activity and mineralisation in mice (Baudry et al., 2010). Hodge et al. (2013) reported the presence of TPH1, SLC6A4, 5HT_1B_, 5HT_2A_ and 5HT_2B_ in mature primary human osteoblasts with the highest expression of TPH_1_. Yun et al. (2016) showed that 5HT_1-7_ expression is higher in bone compared to brain, where the 5HT_6_ expression was the highest followed by 5HT_2_ in primary mice calvarial osteoblasts where 5HT_6_ inhibited osteoblast differentiation via Jab1 in BMP2 signalling but not PKA and ERK1/2 (Yun et al., 2016). This data suggest the possible role of 5HT_6_ in regulating abnormal osteoblasts differentiation.

In summary, expression of serotonin receptors has been clearly demonstrated in both animal and human models of osteoclast- and osteoblastogenesis. Receptors 5HT_1B_, 5HT_2A_, 5HT_2B_ and 5HT_4_ are found to be implicated in regulating osteoclast and osteoblast activity and more specifically the loss of 5HT_2B_ leads to decreased bone formation (Battaglino et al., 2004; Collet et al., 2008; Baudry et al., 2010; Chabbi-Achengli et al., 2012; Hodge et al., 2013; Park et al., 2018) while 5HT_6_ may important in regulating osteoblasts differentiation (Yun et al., 2016). There are relatively few reports available, however, that detail associated signalling pathways downstream of serotonin receptor binding in bone cell function, especially in human models. Therefore, it is important to further investigate serotonin receptor expression profile in human bone cells to better understand antipsychotic-induced bone loss as SGA and TGA have varying affinities to these receptors.

## 7 Antipsychotics and bone cell function: importance of adrenergic receptors/signalling in osteoclasts and osteoblasts

Adrenergic receptors (adrenoceptors) are GPCRs that are involved in activating neurotransmitters; noradrenaline (norepinephrine) and adrenaline (epinephrine) (Graham, 1990). These receptors are expressed in the peripheral and central nervous system as hetero receptors inhibiting the release of neurotransmitters such as serotonin, gamma-aminobutyric acid (GABA) and dopamine (Cruz Grecco Teixeira et al., 2016). There are two main types of adrenergic receptors; alpha (α) and beta (β), with multiple subtypes. Alpha receptors have subtypes α1 (Adra1) and α2 (Adra2) with further sub-categories α1A (Adra1a), α1B (Adra1b), α1D (Adra1d), and α2A (Adra2a), α2B (Adra2b), α2C (Adra2c) (Perez, 2020) while beta receptors have subtypes β1 (Adrb1), β2 (Adrb2) and β3 (Adrb3). The Adra1, Adra2 and Adrb modulate signalling pathways through G_q/11_, G_i_ and G_s_, respectively (Figure 2) (Bylund et al., 2013).

### 7.1 Adrenergic signalling in osteoclasts

Mammalian bone is innervated by sympathetic nerves (Hill and Elde, 1991; Khosla et al., 2018). In normal bone remodelling, noradrenaline is released by sympathetic nerves, which are known to inhibit bone formation and stimulate bone resorption (Khosla et al., 2018). Adrenergic receptor expression has been shown in human osteoclasts and osteoblasts (Table 4). The expression of β-adrenergic receptors have been demonstrated in both osteoclasts and osteoblasts (Kondo et al., 2013). Activation of β-adrenergic receptors regulate the secretion of cytokines by osteoblasts, which indirectly stimulated osteoclast activity in human bone marrow-derived osteoclast-like mononuclear cells (Arai et al., 2003; Zhong and Xia, 2021). Activation of Adrb2 inhibited CREB phosphorylation and subsequent osteoblast proliferation, which result in increased ATF4 phosphorylation and RANKL expression leading to increased osteoclast activity and bone resorption (Elenkov et al., 2001; Kajimura et al., 2011; Zhong and Xia, 2021). It has been suggested that β–adrenergic receptor stimulation may also increase RANKL production by T cells, which also may drive osteoclastogenesis (Elenkov et al., 2001; Bonnet et al., 2008).

**TABLE 4:** Adrenergic receptor expression in osteoclasts and osteoblasts.

Type of adrenergic receptor	Experiment cell type	References
Osteoclasts (OC)		
Adra1a, Adra2a Adra2b and Adra2c	RAW 264.7 cells	Suga et al. (2010), Hamajima et al. (2018)
Adrb2 in mature OC	BM cells collected from male C57BL/6J mice and RAW264.7 cells	Kondo et al. (2013)
Adra2a, Adra2b and Adra2c	Primary BM cells from C57BL/6J mice	Hamajima et al. (2018)
Adra1b, Adra2b and Adrb2	Human BM derived OC- like MNCs	Arai et al. (2003)
Adra1b, Adra2b and Adrb2	Human BM derived OC	Togari (2002)
Osteoblasts (OB)		
Adra1b, Adra1d and Adrb2	MC3T3-E1 osteoblastic cell line	Nishiura and Abe (2007), Moriya et al. (2015)
Adrb1, Adrb2 and Adrb3	Primary mice OB	Ma et al. (2011)
Adrb1, Adrb2 and Adrb3	Female Sprague-Dawley rat condylar subchondral bone	Jiao et al. (2015)
Adra1b, Adrb1, Adrb2 and Adrb3	Human SaM1, SaOS2, HOS and MG63	Togari et al. (1997), Togari (2002)
Adrb1 and Adrb2	Human OB cell line	Khosla et al. (2018)
Adra1b, Adra2a and Adrb2	Trabecular ends of human fetal long bone and MG63 cells	Huang et al. (2009)

Animal studies have shown that β-receptor agonists increase the levels of prostaglandin 2 and interleukin-6 (IL-6), which promotes osteoclast proliferation and resorption in mice skulls, thereby decreasing bone mass (Zhong and Xia, 2021). The mouse osteoclast precursor cell line RAW264.7 expresses Adrb2 and these receptors stimulate osteoclastogenesis via reactive oxygen species production (Zhong and Xia, 2021). The presence of all three Adra2 receptors in RAW264.7 cells have been reported on days 0, 2 and 4 after administration of RANKL while there was no significant difference in receptor expression between days 0, 2 and 4 (Hamajima et al., 2018). In contrast, in mice primary bone marrow cells, Adra2b showed to be upregulated after RANKL administration while Adra2a and Adra2c expression was significantly downregulated (Hamajima et al., 2018). Similarly, expression of Adrb2, but not Adrb1 and Adrb3, is detected in mature osteoclasts derived from mice BMSCs (Zhong and Xia, 2021). It has also been reported that Adra2 agonists suppress osteoclastic gene expression in RAW264.7 cells and in primary bone marrow cells, namely; NFATc1, tartrate-resistant acid phosphatase (TRAP) and cathepsin K, with a corresponding decrease in TRAP positive multi-nucleated osteoclast number in mouse BMSCs (Hamajima et al., 2018).

### 7.2 Adrenergic signalling in osteoblasts

Receptor Adrb2 is thought to be the main receptor expressed in sympathetic nerves responsible for the regulation of bone mass via osteoblasts (Zhong and Xia, 2021). Adrb2 negatively impacts bone formation by inhibiting the cAMP/CREB pathway, which is important in osteoblast activity (Dimitri and Rosen, 2017; Zhong and Xia, 2021). It has also been shown that parathyroid hormone (PTH) suppresses Adrb2 expression in the MC3T3-E1 osteoblastic cell line mediating phosphorylation of CREB (Moriya et al., 2015). In bone cells, the major pathway involved in PTH is via cAMP/PKA/CREB where the phosphorylation of CREB upregulates osteoblasts differentiation and bone formation (Zhang et al., 2011). Moreover, in animal studies, Adrb2-deficient mice have shown higher osteoblast activity and high bone mass with decreased resorption (Takeda et al., 2002; Elefteriou et al., 2005; Zhong and Xia, 2021). Other studies have also shown that blocking Adrb2 increases bone mass (Pasco et al., 2004; Sato et al., 2010).

Receptors Adra1b and Adra1d was strongly expressed in MC3T3-E1 cells (Nishiura and Abe, 2007). Likewise, Adra1b and Adrb2 were detected in human osteoblasts and MG63 cells, whereas expression of Adra2a was relatively weak, with low levels of expression of Adra2c only in demonstrated in MG63 cells (Huang et al., 2009). This study also showed the expression of Adra1b to be three-fold higher than Adrb2 expression in human osteoblasts. It has been reported the expression of Adrb1, Adrb2, Adrb3 and Adra1b receptors in human periosteum-derived osteoblastic cells (SaM1) and human osteosarcoma-derived cells: SaOS2, HOS and MG63 (Togari et al., 1997; Togari, 2002).

In human osteoblasts, both Adra1 and Adrb2 agonists stimulate RANKL and soluble decoy RANK receptor osteoprotegerin (OPG) mRNA expression, where Adra1 agonists stimulated proliferation and OPG mRNA expression at lower concentration than required for RANKL stimulation. The Adrb2 agonists predominantly upregulated resorption signals by increasing RANKL expression at lower concentration than required for OPG stimulation. Moreover, beta blockers inhibited Adrb2 induced RANKL mRNA expression, but not OPG mRNA expression (Huang et al., 2009), which could result in higher BMD and protection against fracture, which has also been shown by Pasco et al. (2004) (Pasco et al., 2004). This suggests the possibility that Adra1 and Adrb2 may be intimately involved in regulating bone turnover. It has also suggested that the PKC and ERK signalling pathways may mediate RANKL expression via Adra1 receptors (Huang et al., 2009).

There is a lack of data on adrenergic receptor expression profile in bone cells, particularly in primary human cells, where adrenergic receptors, especially Adrb2, are associated with bone mass regulation. In summary, both osteoclasts and osteoblasts express adrenergic receptors including Adra1b, Adra2a, Adra2b, Adra2c, Adra1d, Adrb1, Adrb2 and Adrb3 (Table 1). Among these receptors, Adrb2 is highly implicated in regulating bone mass, where Adrb2-signaling increases resorption in osteoclasts via prostaglandin 2 and IL-6, while decreasing bone formation via cAMP/PKA/CREB/AP1 signalling leading to overall decrease in bone mass (Kellenberger et al., 1998; Arai et al., 2003; Zhong and Xia, 2021). Moreover, Adra1 also regulates osteoclast resorption via PKC/ERK pathway (Huang et al., 2009).

## 8 Conclusion

In light of the emerging clinical evidence that schizophrenia and antipsychotics both independently decrease BMD and increase fracture risk, there remains a need to build on the existing evidence identifying the presence of dopamine, serotonin and adrenergic receptors in bone. The evidence to date indicate that dopamine directly inhibits osteoclastogenesis via the D_2_/cAMP/PKA/CREB pathway (Wang et al., 2021b). In contrast, dopamine stimulates osteoblastogenesis via the D_1_/ERK 1 and 2/Runx2 pathway while inhibition of cAMP/PKA can inhibit dopamine-induced osteogenesis (Wang et al., 2020). Centrally-produced serotonin decreases bone resorption and increases bone formation. However, in the periphery, serotonin directly acts on osteoblasts to inhibit bone formation (Ducy and Karsenty, 2010). Data indicate that 5HT_2B_ and 5HT_6_ play an important role in maintaining bone mass. It has been suggested that bone turnover is regulated by Adrb2 and Adra1 by regulating RANKL and OPG levels (Huang et al., 2009). Further, activation of Adrb2 inhibits CREB phosphorylation leading to decreased osteoblast proliferation and Adrb2 can activate cAMP/PKA/AP1 pathways to regulate bone formation (Kellenberger et al., 1998; Arai et al., 2003; Zhong and Xia, 2021).

Therefore, we assume that antipsychotics may cause imbalance in bone formation and resorption via dopamine, serotonin and adrenergic receptors through cAMP/PKA/CREB, ERK 1/2, AP1 and Jab1/Smad1, 5, 8/BMP2 pathways. Further studies are required in animal and human models of bone formation and resorption to help characterise the role of associated signalling pathways and the direct impact of antipsychotics on different receptors present in bone cells. Given antipsychotics largely target dopamine and serotonin receptors, as well as, albeit to a lesser extent adrenergic receptors, it is important to explore the mechanism(s) of action by which antipsychotics impact bone loss.

## References

[B1] Abbas A. I. Hedlund P. B. Huang X. P. Tran T. B. Meltzer H. Y. Roth B. L. (2009). Amisulpride is a potent 5-HT7 antagonist: Relevance for antidepressant actions *in vivo* . Psychopharmacol. Berl. 205 (1), 119–128. 10.1007/s00213-009-1521-8 PMC2821721 19337725

[B2] Abu-Amer Y. (2013). NF-κB signaling and bone resorption. Osteoporos. Int. 24 (9), 2377–2386. 10.1007/s00198-013-2313-x 23468073 PMC3884829

[B3] Al Shirawi M. I. Edgar N. E. Kennedy S. H. (2017). Brexpiprazole in the treatment of major depressive disorder. Clin. Med. Insights Ther. 9, 1179559X1773180. 10.1177/1179559x17731801

[B4] Arai M. Nagasawa T. Koshihara Y. Yamamoto S. Togari A. (2003). Effects of β-adrenergic agonists on bone-resorbing activity in human osteoclast-like cells. Biochimica Biophysica Acta (BBA) - Mol. Cell Res. 1640 (2), 137–142. 10.1016/s0167-4889(03)00042-9 12729923

[B5] Aringhieri S. Carli M. Kolachalam S. Verdesca V. Cini E. Rossi M. (2018). Molecular targets of atypical antipsychotics: From mechanism of action to clinical differences. Pharmacol. Ther. 192, 20–41. 10.1016/j.pharmthera.2018.06.012 29953902

[B6] Arnaiz J. A. Rodrigues-Silva C. Mezquida G. Amoretti S. Cuesta M. J. Fraguas D. (2021). The usefulness of Olanzapine plasma concentrations in monitoring treatment efficacy and metabolic disturbances in first-episode psychosis. Psychopharmacol. Berl. 238 (3), 665–676. 10.1007/s00213-020-05715-5 33230696

[B7] Asano T. Tanaka K.-I. Tada A. Shimamura H. Tanaka R. Maruoka H. (2017). Ameliorative effect of chlorpromazine hydrochloride on visceral hypersensitivity in rats: Possible involvement of 5-ht2a receptor. Br. J. Pharmacol. 174 (19), 3370–3381. 10.1111/bph.13960 28750135 PMC5595764

[B181] Australian Institute of Health and Welfare (2022). Mental health-related prescriptions. Available from: https://www.aihw.gov.au/reports/mental-health-services/mental-health-services-in-australia/report-content/mental-health-related-prescriptions. (Accessed October 19, 2022).

[B8] Azimi Manavi B. Stuart A. L. Pasco J. A. Hodge J. M. Samarasinghe R. M. Weerasinghe D. K. (2023). Use of antipsychotic medication and its relationship with bone mineral density: A population-based study of men and women. Front. Psychiatry 13, 13. 10.3389/fpsyt.2022.1004366 PMC9849889 36684026

[B9] Ban T. A. (2007). Fifty years chlorpromazine: A historical perspective. Neuropsychiatr. Dis. Treat. 3 (4), 495–500. 19300578 PMC2655089

[B10] Battaglino R. Fu J. Späte U. Ersoy U. Joe M. Sedaghat L. (2004). Serotonin regulates osteoclast differentiation through its transporter. J. Bone Mineral Res. 19 (9), 1420–1431. 10.1359/JBMR.040606 15312242

[B11] Baudry A. Bitard J. Mouillet-Richard S. Locker M. Poliard A. Launay J.-M. (2010). Serotonergic 5-HT2B receptor controls tissue-nonspecific alkaline phosphatase activity in osteoblasts via eicosanoids and phosphatidylinositol-specific phospholipase C. J. Biol. Chem. 285 (34), 26066–26073. 10.1074/jbc.M109.073791 20573958 PMC2924006

[B12] Becker D. Liver O. Mester R. Rapoport M. Weizman A. Weiss M. (2003). Risperidone, but not olanzapine, decreases bone mineral density in female premenopausal schizophrenia patients. J. Clin. Psychiatry 64 (7), 761–766. 10.4088/jcp.v64n0704 12934975

[B13] Berger M. Gray J. A. Roth B. L. (2009). The expanded biology of serotonin. Annu. Rev. Med. 60, 355–366. 10.1146/annurev.med.60.042307.110802 19630576 PMC5864293

[B14] Bliziotes M. Eshleman A. Burt-Pichat B. Zhang X. W. Hashimoto J. Wiren K. (2006). Serotonin transporter and receptor expression in osteocytic MLO-Y4 cells. Bone 39 (6), 1313–1321. 10.1016/j.bone.2006.06.009 16884969 PMC1766480

[B15] Bliziotes M. M. Eshleman A. J. Zhang X. W. Wiren K. M. (2001). Neurotransmitter action in osteoblasts: Expression of a functional system for serotonin receptor activation and reuptake. Bone 29 (5), 477–486. 10.1016/s8756-3282(01)00593-2 11704501

[B16] Boer M. Castelein S. Wiersma D. Schoevers R. Knegtering R. (2015). The facts about sexual (Dys)function in schizophrenia: An overview of clinically relevant findings. Schizophr. Bull. 41, 674–686. 10.1093/schbul/sbv001 25721311 PMC4393701

[B17] Bolton J. M. Morin S. N. Majumdar S. R. Sareen J. Lix L. M. Johansson H. (2017). Association of mental disorders and related medication use with risk for major osteoporotic fractures. JAMA Psychiatry 74 (6), 641–648. 10.1001/jamapsychiatry.2017.0449 28423154 PMC5539842

[B18] Bonnet N. Pierroz D. D. Ferrari S. L. (2008). Adrenergic control of bone remodeling and its implications for the treatment of osteoporosis. J. Musculoskelet. Neuronal Interact. 8 (2), 94–104. 18622078

[B19] Brenner B. Harney J. T. Ahmed B. A. Jeffus B. C. Unal R. Mehta J. L. (2007). Plasma serotonin levels and the platelet serotonin transporter. J. Neurochem. 102 (1), 206–215. 10.1111/j.1471-4159.2007.04542.x 17506858 PMC3041643

[B20] Bylund D. B. (2013). “Adrenergic receptors,” in Encyclopedia of biological chemistry. Editors Lennarz W. J. Lane M. D. Second Edition (Waltham: Academic Press), 57–60.

[B21] Calarge C. A. Zimmerman B. Xie D. Kuperman S. Schlechte J. A. (2010). A cross-sectional evaluation of the effect of risperidone and selective serotonin reuptake inhibitors on bone mineral density in boys. J. Clin. Psychiatry 71 (3), 338–347. 10.4088/JCP.08m04595gre 20331935 PMC2845988

[B22] Caplan A. I. (1991). Mesenchymal stem cells. J. Orthop. Res. 9 (5), 641–650. 10.1002/jor.1100090504 1870029

[B23] Chabbi-Achengli Y. Coudert Amélie E. Callebert J. Geoffroy V. Côté F. Collet C. (2012). Decreased osteoclastogenesis in serotonin-deficient mice. Proc. Natl. Acad. Sci. 109 (7), 2567–2572. 10.1073/pnas.1117792109 22308416 PMC3289318

[B24] Chen H. Tan X.-N. Hu S. Liu R.-Q. Peng L.-H. Li Y.-M. (2021). Molecular mechanisms of chondrocyte proliferation and differentiation. Front. Cell Dev. Biol. 9. 10.3389/fcell.2021.664168 PMC8194090 34124045

[B25] Cheong P.-U. Ma T. Zheng Y. Ge X.-Y. Zhang Y. Lin Y. (2018). Dopamine receptor expression on primary osteoblasts and bone marrow mesenchymal stem cells of rats. Int. J. Clin. Exp. Med. 11 (3), 1765–1771.

[B26] Chetty M. Miller R. (1991). Effect of storage on the plasma concentration of chlorpromazine and six of its metabolites. Ther. Drug Monit. 13 (4), 350–355. 10.1097/00007691-199107000-00012 1780969

[B27] Ching-Min K. Wei-Jen L. Chun-Che H. Tsuo-Hung L. Ching-Heng L. Shun-Ping W. (2020). Antipsychotic medication in schizophrenic patients is associated with higher risks of developing bone fractures and refractures. Clin. Psychopharmacol. Neurosci. 18 (4), 562–570. 10.9758/cpn.2020.18.4.562 33124588 PMC7609221

[B28] Clapham E. Boden R. Reutfors J. Svensson T. Ramcharran D. Qiu H. (2020). Exposure to risperidone versus other antipsychotics and risk of osteoporosis-related fractures: A population-based study. Acta Psychiatr. Scand. 141 (1), 74–83. 10.1111/acps.13101 31545521 PMC6973241

[B29] ClinicalTrials.gov (2014). Safety, tolerability, and pharmacokinetics of iloperidone depot in schizophrenic patients ClinicalTrials.gov2014. Available from: https://clinicaltrials.gov/ct2/show/NCT01348100 .

[B30] Coentre R. Barrocas D. Levy P. (2009). Low bone mineral density and psychosis: A multifactorial relation. Eur. Psychiatry 24 (S1), 1. 10.1016/s0924-9338(09)70491-2 18926669

[B31] Cohen J. Y. Dumoulin-Charette A. Meraabi N. Poirier R. (2019). Serum concentration of paliperidone palmitate administered every 3 weeks. Psychopharmacol. Bull. 49 (2), 57–62. 31308584 10.64719/pb.4595 PMC6598777

[B32] Collet C. Schiltz C. Geoffroy V. Maroteaux L. Launay J. M. de Vernejoul M. C. (2008). The serotonin 5-HT2B receptor controls bone mass via osteoblast recruitment and proliferation. Faseb J. 22 (2), 418–427. 10.1096/fj.07-9209com 17846081 PMC5409955

[B33] Cookson J. (2018). Rapid tranquillisation: The science and advice. BJPsych Adv. 24, 346–358. 10.1192/bja.2018.25

[B34] Correll C. U. Koblan K. S. Hopkins S. C. Li Y. Heather D. Goldman R. (2021). Safety and effectiveness of ulotaront (SEP-363856) in schizophrenia: Results of a 6-month, open-label extension study. npj Schizophr. 7 (1), 63. 10.1038/s41537-021-00190-z 34887427 PMC8660889

[B35] Coryell W. Miller D. D. Perry P. J. (1998). Haloperidol plasma levels and dose optimization. Am. J. Psychiatry 155 (1), 48–53. 10.1176/ajp.155.1.48 9433338

[B36] Costa J. L. Smith G. Watson M. Lin J.-M. Callon K. Gamble G. (2011). The atypical anti-psychotic clozapine decreases bone mass in rats *in vivo* . Schizophrenia Res. 126 (1), 291–297. 10.1016/j.schres.2010.11.024 21185156

[B37] Crews M. P. Howes O. D. (2012). Is antipsychotic treatment linked to low bone mineral density and osteoporosis? A review of the evidence and the clinical implications. Hum. Psychopharmacol. 27 (1), 15–23. 10.1002/hup.1265 22228316 PMC3731625

[B38] Cruz Grecco Teixeira M. B. Martins G. M. Miranda-Rodrigues M. De Araújo I. F. Oliveira R. Brum P. C. (2016). Lack of α2C-adrenoceptor results in contrasting phenotypes of long bones and vertebra and prevents the thyrotoxicosis-induced osteopenia. PLoS One 11 (1), e0146795–e. 10.1371/journal.pone.0146795 26815679 PMC4729682

[B39] Dai S. Q. Yu L. P. Shi X. Wu H. Shao P. Yin G. Y. (2014). Serotonin regulates osteoblast proliferation and function *in vitro* . Braz J. Med. Biol. Res. 47 (9), 759–765. 10.1590/1414-431x20143565 25098615 PMC4143203

[B40] de Bartolomeis A. Tomasetti C. Iasevoli F. (2015). Update on the mechanism of action of aripiprazole: Translational insights into antipsychotic strategies beyond dopamine receptor antagonism. CNS Drugs 29 (9), 773–799. 10.1007/s40263-015-0278-3 26346901 PMC4602118

[B41] De Hert M. Detraux J. van Winkel R. Yu W. Correll C. U. (2011). Metabolic and cardiovascular adverse effects associated with antipsychotic drugs. Nat. Rev. Endocrinol. 8 (2), 114–126. 10.1038/nrendo.2011.156 22009159

[B42] Del'Guidice T. Lemasson M. Beaulieu J.-M. (2011). Role of beta-arrestin 2 downstream of dopamine receptors in the basal ganglia. Front. Neuroanat. 5, 58. 10.3389/fnana.2011.00058 21922001 PMC3167352

[B43] Dimitri P. Rosen C. (2017). The central nervous system and bone metabolism: An evolving story. Calcif. Tissue Int. 100 (5), 476–485. 10.1007/s00223-016-0179-6 27501818

[B44] Ducy P. Karsenty G. (2010). The two faces of serotonin in bone biology. J. Cell Biol. 191 (1), 7–13. 10.1083/jcb.201006123 20921133 PMC2953449

[B45] El Solh A. (2018). Management of nightmares in patients with posttraumatic stress disorder: Current perspectives. Nat. Sci. Sleep 10, 409–420. 10.2147/NSS.S166089 30538593 PMC6263296

[B46] El-Merahbi R. Löffler M. Mayer A. Sumara G. (2015). The roles of peripheral serotonin in metabolic homeostasis. FEBS Lett. 589 (15), 1728–1734. 10.1016/j.febslet.2015.05.054 26070423

[B47] Elefteriou F. Ahn J. D. Takeda S. Starbuck M. Yang X. Liu X. (2005). Leptin regulation of bone resorption by the sympathetic nervous system and CART. Nature 434 (7032), 514–520. 10.1038/nature03398 15724149

[B48] Elenkov I. J. Wilder R. Chrousos G. Vizi E. (2001). The sympathetic nerve—an integrative interface between two supersystems: The brain and the immune system. Pharmacol. Rev. 52, 595–638. 11121511

[B49] Florencio-Silva R. Sasso G. RdS. Sasso-Cerri E. Simões M. J. Cerri P. S. (2015). Biology of bone tissue: Structure, function, and factors that influence bone cells. Biomed. Res. Int. 2015, 421746. 10.1155/2015/421746 26247020 PMC4515490

[B50] Foley P. Gerlach M. Double K. Riederer P. (2004). Dopamine receptor agonists in the therapy of Parkinson’s disease. Acta Neuroveg. 111, 1375–1446. 10.1007/s00702-003-0059-x 15480844

[B51] Forns J. Layton J. B. Bartsch J. Turner M. E. Dempsey C. Anthony M. (2021). Increased risk of falls and fractures in patients with psychosis and Parkinson disease. PLoS One 16 (1), e0246121. 10.1371/journal.pone.0246121 33503061 PMC7840029

[B52] Frankel J. S. Schwartz T. L. (2017). Brexpiprazole and cariprazine: Distinguishing two new atypical antipsychotics from the original dopamine stabilizer aripiprazole. Ther. Adv. Psychopharmacol. 7 (1), 29–41. 10.1177/2045125316672136 28101322 PMC5228714

[B53] Fraser L.-A. Liu K. Naylor K. L. Hwang Y. J. Dixon S. N. Shariff S. Z. (2015). Falls and fractures with atypical antipsychotic medication use: A population-based cohort study. JAMA Intern. Med. 175 (3), 450–452. 10.1001/jamainternmed.2014.6930 25581312

[B54] Gaskill P. J. Carvallo L. Eugenin E. A. Berman J. W. (2012). Characterization and function of the human macrophage dopaminergic system: Implications for CNS disease and drug abuse. J. Neuroinflammation 9 (1), 203. 10.1186/1742-2094-9-203 22901451 PMC3488577

[B55] Gesty-Palmer D. Flannery P. Yuan L. Corsino L. Spurney R. Lefkowitz R. J. (2009). A beta-arrestin-biased agonist of the parathyroid hormone receptor (PTH1R) promotes bone formation independent of G protein activation. Sci. Transl. Med. 1 (1), 1ra1. 10.1126/scitranslmed.3000071 PMC2852200 20368153

[B56] Graham R. M. (1990). Adrenergic receptors: Structure and function. Clevel. Clin. J. Med. 57 (5), 481–491. 10.3949/ccjm.57.5.481 2164898

[B57] Gustafsson B. I. Thommesen L. Stunes A. K. Tommeras K. Westbroek I. Waldum H. L. (2006). Serotonin and fluoxetine modulate bone cell function *in vitro* . J. Cell Biochem. 98 (1), 139–151. 10.1002/jcb.20734 16408289

[B58] Gustafsson B. I. Westbroek I. Waarsing J. H. Waldum H. Solligård E. Brunsvik A. (2006). Long-term serotonin administration leads to higher bone mineral density, affects bone architecture, and leads to higher femoral bone stiffness in rats. J. Cell Biochem. 97 (6), 1283–1291. 10.1002/jcb.20733 16329113

[B59] Haddad P. M. Correll C. U. (2018). The acute efficacy of antipsychotics in schizophrenia: A review of recent meta-analyses. Ther. Adv. Psychopharmacol. 8 (11), 303–318. 10.1177/2045125318781475 30344997 PMC6180374

[B60] Hálfdánarson Ó. Zoëga H. Aagaard L. Bernardo M. Brandt L. Fusté A. C. (2017). International trends in antipsychotic use: A study in 16 countries, 2005-2014. Eur. Neuropsychopharmacol. 27 (10), 1064–1076. 10.1016/j.euroneuro.2017.07.001 28755801

[B61] Hamajima K. Hamamura K. Chen A. Yokota H. Mori H. Yo S. (2018). Suppression of osteoclastogenesis via α2-adrenergic receptors. Biomed. Rep. 8 (5), 407–416. 10.3892/br.2018.1075 29725523 PMC5920467

[B62] Hanami K. Nakano K. Saito K. Okada Y. Yamaoka K. Kubo S. (2013). Dopamine D2-like receptor signaling suppresses human osteoclastogenesis. Bone 56 (1), 1–8. 10.1016/j.bone.2013.04.019 23631878

[B63] Handa K. Kiyohara S. Yamakawa T. Ishikawa K. Hosonuma M. Sakai N. (2019). Bone loss caused by dopaminergic degeneration and levodopa treatment in Parkinson's disease model mice. Sci. Rep. 9 (1), 13768. 10.1038/s41598-019-50336-4 31551490 PMC6760231

[B64] Handley S. A. Bowskill S. V. J. Patel M. X. Flanagan R. J. (2013). Plasma quetiapine in relation to prescribed dose and other factors: Data from a therapeutic drug monitoring service, 2000-2011. Ther. Adv. Psychopharmacol. 3 (3), 129–137. 10.1177/2045125312470677 24167685 PMC3805454

[B65] Hattori N. Hasegawa K. Sakamoto T. (2012). Pharmacokinetics and effect of food after oral administration of prolonged-release tablets of ropinirole hydrochloride in Japanese patients with Parkinson's disease. J. Clin. Pharm. Ther. 37 (5), 571–577. 10.1111/j.1365-2710.2012.01336.x 22390368

[B66] Hill E. L. Elde R. (1991). Distribution of CGRP-VIP-D beta H-SP-and NPY-immunoreactive nerves in the periosteum of the rat. Cell Tissue Res. 264 (3), 469–480. 10.1007/BF00319037 1714353

[B67] Hioki T. Kuroyanagi G. Matsushima-Nishiwaki R. Kozawa O. Tokuda H. (2022). Oncostatin M attenuates tumor necrosis factor-α-induced synthesis of macrophage-colony stimulating factor via suppression of Akt in osteoblasts. Connect. Tissue Res., 1–9. 10.1080/03008207.2022.2109468 35986560

[B68] Hodge J. M. Wang Y. Berk M. Collier F. M. Fernandes T. J. Constable M. J. (2013). Selective serotonin reuptake inhibitors inhibit human osteoclast and osteoblast formation and function. Biol. Psychiatry 74 (1), 32–39. 10.1016/j.biopsych.2012.11.003 23260229

[B69] Hornykiewicz O. (2017). L-DOPA. J. Park. Dis. 7 (1), S3–S10. 10.3233/jpd-179004 PMC5345651 28282813

[B70] Houseknecht K. L. Bouchard C. C. Black C. A. (2017). Elucidating the mechanism(s) underlying antipsychotic and antidepressant-mediated fractures. J. Ment. Health Clin. Psychol. 1 (1), 9–13. 10.29245/2578-2959/2018/1.1106 31008454 PMC6469345

[B71] Howes O. D. Egerton A. Allan V. McGuire P. Stokes P. Kapur S. (2009). Mechanisms underlying psychosis and antipsychotic treatment response in schizophrenia: Insights from PET and SPECT imaging. Curr. Pharm. Des. 15 (22), 2550–2559. 10.2174/138161209788957528 19689327 PMC3687204

[B72] Huang H. H. Brennan T. C. Muir M. M. Mason R. S. (2009). Functional alpha1-and beta2-adrenergic receptors in human osteoblasts. J. Cell. Physiology 220 (1), 267–275. 10.1002/jcp.21761 19334040

[B73] Ishibashi T. Horisawa T. Tokuda K. Ishiyama T. Ogasa M. Tagashira R. (2010). Pharmacological profile of lurasidone, a novel antipsychotic agent with potent 5-hydroxytryptamine 7 (5-HT7) and 5-HT1A receptor activity. J. Pharmacol. Exp. Ther. 334 (1), 171–181. 10.1124/jpet.110.167346 20404009

[B74] Jiao K. Niu L.-N. Li Q.-H. Ren G.-T. Zhao C.-M. Liu Y.-D. (2015). β2-adrenergic signal transduction plays a detrimental role in subchondral bone loss of temporomandibular joint in osteoarthritis. Sci. Rep. 5 (1), 12593. 10.1038/srep12593 26219508 PMC4518212

[B75] Jörg M. Scammells P. J. Capuano B. Kaczor A. A. Poso A. Mak F. S. (2014). Investigation of novel ropinirole analogues: Synthesis, pharmacological evaluation and computational analysis of dopamine D2 receptor functionalized congeners and homobivalent ligands. MedChemComm 5 (7), 891–898. 10.1039/c4md00066h

[B76] Jung D.-U. Kelly D. L. Oh M.-K. Kong B.-G. Kang J.-W. Lee S.-J. (2011). Bone mineral density and osteoporosis risk in older patients with schizophrenia. J. Clin. Psychopharmacol. 31 (4), 406–410. 10.1097/JCP.0b013e318221b123 21694624

[B77] Kajimura D. Hinoi E. Ferron M. Kode A. Riley K. J. Zhou B. (2011). Genetic determination of the cellular basis of the sympathetic regulation of bone mass accrual. J. Exp. Med. 208 (4), 841–851. 10.1084/jem.20102608 21444660 PMC3135354

[B78] Kanova M. Kohout P. (2021). Serotonin-its synthesis and roles in the healthy and the critically ill. Int. J. Mol. Sci. 22 (9), 4837. 10.3390/ijms22094837 34063611 PMC8124334

[B79] Kawamura H. Arai M. Togari A. (2011). Inhibitory effect of chlorpromazine on RANKL-induced osteoclastogenesis in mouse bone marrow cells. J. Pharmacol. Sci. 117 (1), 54–62. 10.1254/jphs.11006fp 21869564

[B80] Kellenberger S. Muller K. Richener H. Bilbe G. (1998). Formoterol and isoproterenol induce c-fos gene expression in osteoblast-like cells by activating beta2-adrenergic receptors. Bone 22 (5), 471–478. 10.1016/s8756-3282(98)00026-x 9600780

[B81] Kenkre J. S. Bassett J. (2018). The bone remodelling cycle. Ann. Clin. Biochem. 55 (3), 308–327. 10.1177/0004563218759371 29368538

[B82] Khosla S. Drake M. T. Volkman T. L. Thicke B. S. Achenbach S. J. Atkinson E. J. (2018). Sympathetic β1-adrenergic signaling contributes to regulation of human bone metabolism. J. Clin. Investigation 128 (11), 4832–4842. 10.1172/JCI122151 PMC6205387 30153111

[B83] Kim J. M. Yang Y. S. Park K. H. Oh H. Greenblatt M. B. Shim J. H. (2019). The ERK MAPK pathway is essential for skeletal development and homeostasis. Int. J. Mol. Sci. 20 (8), 1803. 10.3390/ijms20081803 31013682 PMC6514701

[B84] Kishimoto T. Watanabe K. Shimada N. Makita K. Yagi G. Kashima H. (2008). Antipsychotic-induced hyperprolactinemia inhibits the hypothalamo-pituitary-gonadal axis and reduces bone mineral density in male patients with schizophrenia. J. Clin. Psychiatry 69 (3), 385–391. 10.4088/jcp.v69n0307 18278991

[B85] Kiss B. Horváth A. Némethy Z. Schmidt E. Laszlovszky I. Bugovics G. (2010). Cariprazine (RGH-188), a dopamine D(3) receptor-preferring, D(3)/D(2) dopamine receptor antagonist-partial agonist antipsychotic candidate: *In vitro* and neurochemical profile. J. Pharmacol. Exp. Ther. 333 (1), 328–340. 10.1124/jpet.109.160432 20093397

[B86] Kiss B. Krámos B. Laszlovszky I. (2022). Potential mechanisms for why not all antipsychotics are able to occupy dopamine D3 receptors in the brain *in vivo* . Front. Psychiatry 13, 785592. 10.3389/fpsyt.2022.785592 35401257 PMC8987915

[B87] Kiss B. Némethy Z. Fazekas K. Kurkó D. Gyertyán I. Sághy K. (2019). Preclinical pharmacodynamic and pharmacokinetic characterization of the major metabolites of cariprazine. Drug Des. Devel Ther. 13, 3229–3248. 10.2147/DDDT.S188760 PMC6754336 31571826

[B88] Kode A. Mosialou I. Silva B. C. Rached M. T. Zhou B. Wang J. (2012). FOXO1 orchestrates the bone-suppressing function of gut-derived serotonin. J. Clin. Invest. 122 (10), 3490–3503. 10.1172/JCI64906 22945629 PMC3461930

[B89] Kondo H. Takeuchi S. Togari A. (2013). β-Adrenergic signaling stimulates osteoclastogenesis via reactive oxygen species. Am. J. Physiol. Endocrinol. Metab. 304 (5), E507–E515. 10.1152/ajpendo.00191.2012 23169789

[B90] Kunimatsu T. Kimura J. Funabashi H. Inoue T. Seki T. (2010). The antipsychotics haloperidol and chlorpromazine increase bone metabolism and induce osteopenia in female rats. Regul. Toxicol. Pharmacol. 58 (3), 360–368. 10.1016/j.yrtph.2010.08.001 20709132

[B91] Laborit H. Huguenard P. Alluaume R. (1952). A new vegetative stabilizer; 4560 R.P. Presse Med. 60 (10), 206–208. 14957790

[B92] Lahdelma L. Oja S. Korhonen M. Andersson L. C. (2010). Clozapine is cytotoxic to primary cultures of human bone marrow mesenchymal stromal cells. J. Clin. Psychopharmacol. 30 (4), 461–463. 10.1097/JCP.0b013e3181e6a082 20631565

[B93] Lally J. (2020). Bone health in psychotic disorders, and risk factors for decreased bone health in established and early psychosis. Royal College of Surgeons in Ireland.

[B94] Lally J. MacCabe J. H. (2015). Antipsychotic medication in schizophrenia: A review. Br. Med. Bull. 114 (1), 169–179. 10.1093/bmb/ldv017 25957394

[B95] Lee D. J. Tseng H. C. Wong S. W. Wang Z. Deng M. Ko C.-C. (2015). Dopaminergic effects on *in vitro* osteogenesis. Bone Res. 3 (1), 15020. 10.1038/boneres.2015.20 26558139 PMC4639997

[B96] Lee J. Y. Yang J.-Y. Kim S. W. (2021). Bone lining cells could Be sources of bone marrow adipocytes. Front. Endocrinol. 12, 766254. 10.3389/fendo.2021.766254 PMC8678613 34925236

[B97] Lencesova L. Szadvari I. Babula P. Kubickova J. Chovancova B. Lopusna K. (2017). Disruption of dopamine D1/D2 receptor complex is involved in the function of haloperidol in cardiac H9c2 cells. Life Sci. 191, 186–194. 10.1016/j.lfs.2017.10.026 29054453

[B98] Lesclous P. Guez D. Llorens A. Saffar J. L. (2001). Time-course of mast cell accumulation in rat bone marrow after ovariectomy. Calcif. Tissue Int. 68 (5), 297–303. 10.1007/BF02390837 11683537

[B99] Li P. Snyder G. L. Vanover K. E. (2016). Dopamine targeting drugs for the treatment of schizophrenia: Past, present and future. Curr. Top. Med. Chem. 16 (29), 3385–3403. 10.2174/1568026616666160608084834 27291902 PMC5112764

[B100] Li P. Wang Y. Liu X. Zhou Z. Wang J. Zhou H. (2019). Atypical antipsychotics induce human osteoblasts apoptosis via Wnt/β-catenin signaling. BMC Pharmacol. Toxicol. 20 (1), 10. 10.1186/s40360-019-0287-9 30755277 PMC6373048

[B101] Lieberman J. A. 3rd. (2004). Metabolic changes associated with antipsychotic use. Prim. Care Companion J. Clin. Psychiatry 6 (2), 8–13. 16001095 PMC487012

[B102] Ma Y. Nyman J. S. Tao H. Moss H. H. Yang X. Elefteriou F. (2011). β2-Adrenergic receptor signaling in osteoblasts contributes to the catabolic effect of glucocorticoids on bone. Endocrinology 152 (4), 1412–1422. 10.1210/en.2010-0881 21266510 PMC3060633

[B103] Maeda K. Sugino H. Akazawa H. Amada N. Shimada J. Futamura T. (2014). Brexpiprazole I: *In vitro* and *in vivo* characterization of a novel serotonin-dopamine activity modulator. J. Pharmacol. Exp. Ther. 350 (3), 589–604. 10.1124/jpet.114.213793 24947465

[B104] Mahapatra J. Quraishi S. N. David A. Sampson S. Adams C. E. (2014). Flupenthixol decanoate (depot) for schizophrenia or other similar psychotic disorders. Cochrane Database Syst. Rev. 2014 (6), Cd001470. 10.1002/14651858.CD001470.pub2 24915451 PMC7057031

[B105] Mailman R. B. Murthy V. (2010). Third generation antipsychotic drugs: Partial agonism or receptor functional selectivity? Curr. Pharm. Des. 16 (5), 488–501. 10.2174/138161210790361461 19909227 PMC2958217

[B106] Maramai S. Gemma S. Brogi S. Campiani G. Butini S. Stark H. (2016). Dopamine D3 receptor antagonists as potential therapeutics for the treatment of neurological diseases. Front. Neurosci. 10, 451. 10.3389/fnins.2016.00451 27761108 PMC5050208

[B107] Marino J. Caballero J. (2010). Iloperidone for the treatment of schizophrenia. Ann. Pharmacother. 44, 863–870. 10.1345/aph.1M603 20388862

[B108] Martel J. C. Gatti McArthur S. (2020). Dopamine receptor subtypes, physiology and pharmacology: New ligands and concepts in schizophrenia. Front. Pharmacol. 11, 1003. 10.3389/fphar.2020.01003 32765257 PMC7379027

[B109] Matt S. M. Gaskill P. J. (2020). Where is dopamine and how do immune cells see it?: Dopamine-mediated immune cell function in health and disease. J. Neuroimmune Pharmacol. 15 (1), 114–164. 10.1007/s11481-019-09851-4 31077015 PMC6842680

[B110] Mauri M. C. Paletta S. Maffini M. Colasanti A. Dragogna F. Di Pace C. (2014). Clinical pharmacology of atypical antipsychotics: An update. EXCLI J. 13, 1163–1191. 26417330 PMC4464358

[B111] May M. Beauchemin M. Vary C. Barlow D. Houseknecht K. L. (2019). The antipsychotic medication, risperidone, causes global immunosuppression in healthy mice. PloS one 14 (6), e0218937. 10.1371/journal.pone.0218937 31242264 PMC6594635

[B112] McCorvy J. D. Roth B. L. (2015). Structure and function of serotonin G protein-coupled receptors. Pharmacol. Ther. 150, 129–142. 10.1016/j.pharmthera.2015.01.009 25601315 PMC4414735

[B113] McCutcheon R. Beck K. Jauhar S. Howes O. D. (2018). Defining the locus of dopaminergic dysfunction in schizophrenia: A meta-analysis and test of the mesolimbic hypothesis. Schizophr. Bull. 44 (6), 1301–1311. 10.1093/schbul/sbx180 29301039 PMC5933516

[B114] McCutcheon R. A. Abi-Dargham A. Howes O. D. (2019). Schizophrenia, dopamine and the striatum: From biology to symptoms. Trends Neurosci. 42 (3), 205–220. 10.1016/j.tins.2018.12.004 30621912 PMC6401206

[B115] Meltzer H. Y. (1994). An overview of the mechanism of action of clozapine. J. Clin. Psychiatry 55, 47–52. 7961573

[B116] Meltzer H. Y. Massey B. W. (2011). The role of serotonin receptors in the action of atypical antipsychotic drugs. Curr. Opin. Pharmacol. 11 (1), 59–67. 10.1016/j.coph.2011.02.007 21420906

[B117] Méndez-Ferrer S. Michurina T. V. Ferraro F. Mazloom A. R. Macarthur B. D. Lira S. A. (2010). Mesenchymal and haematopoietic stem cells form a unique bone marrow niche. Nature 466 (7308), 829–834. 10.1038/nature09262 20703299 PMC3146551

[B118] Metzger C. E. Narayanan S. A. (2019). The role of osteocytes in inflammatory bone loss. Front. Endocrinol. 10, 285. 10.3389/fendo.2019.00285 PMC6527760 31139147

[B119] Midha K. K. Korchinski E. D. Verbeeck R. K. Roscoe R. M. Hawes E. M. Cooper J. K. (1983). Kinetics of oral trifluoperazine disposition in man. Br. J. Clin. Pharmacol. 15 (3), 380–382. 10.1111/j.1365-2125.1983.tb01515.x 6849769 PMC1427786

[B120] Miron R. J. Bosshardt D. D. (2016). OsteoMacs: Key players around bone biomaterials. Biomaterials 82, 1–19. 10.1016/j.biomaterials.2015.12.017 26735169

[B121] Mohamad S. F. Gunawan A. Blosser R. Childress P. Aguilar-Perez A. Ghosh J. (2021). Neonatal osteomacs and bone marrow macrophages differ in phenotypic marker expression and function. J. Bone Mineral Res. 36 (8), 1580–1593. 10.1002/jbmr.4314 PMC10229197 33900648

[B122] Möller H. J. (2003). Amisulpride: Limbic specificity and the mechanism of antipsychotic atypicality. Prog. Neuropsychopharmacol. Biol. Psychiatry 27 (7), 1101–1111. 10.1016/j.pnpbp.2003.09.006 14642970

[B123] Moriya S. Hayata T. Notomi T. Aryal S. Nakamaoto T. Izu Y. (2015). PTH regulates β2-adrenergic receptor expression in osteoblast-like mc3t3-E1 cells. J. Cell. Biochem. 116 (1), 142–148. 10.1002/jcb.24953 25164990

[B124] Motyl K. J. Beauchemin M. Barlow D. Le P. T. Nagano K. Treyball A. (2017). A novel role for dopamine signaling in the pathogenesis of bone loss from the atypical antipsychotic drug risperidone in female mice. Bone 103, 168–176. 10.1016/j.bone.2017.07.008 28689816 PMC5573184

[B125] Motyl K. J. Dick-de-Paula I. Maloney A. E. Lotinun S. Bornstein S. de Paula F. J. A. (2012). Trabecular bone loss after administration of the second-generation antipsychotic risperidone is independent of weight gain. Bone 50 (2), 490–498. 10.1016/j.bone.2011.08.005 21854880 PMC3261344

[B126] Nagasawa S. Yamaguchi R. Saka K. Torimitsu S. Chiba F. Yajima D. (2022). Ropinirole involved in a fatal case: Blood and urinary concentrations. Forensic Toxicol. 40 (1), 173–179. 10.1007/s11419-021-00593-8 36454487

[B127] Nakashioya H. Nakano K. Watanabe N. Miyasaka N. Matsushita S. Kohsaka H. (2011). Therapeutic effect of D1-like dopamine receptor antagonist on collagen-induced arthritis of mice. Mod. Rheumatol. 21 (3), 260–266. 10.1007/s10165-010-0387-2 21188452

[B128] Neve K. A. Seamans J. K. Trantham-Davidson H. (2004). Dopamine receptor signaling. J. Recept. Signal Transduct. 24 (3), 165–205. 10.1081/rrs-200029981 15521361

[B129] Nichols D. E. Nichols C. D. (2008). Serotonin receptors. Chem. Rev. 108 (5), 1614–1641. 10.1021/cr078224o 18476671

[B130] Nishiura T. Abe K. (2007). Alpha1-adrenergic receptor stimulation induces the expression of receptor activator of nuclear factor kappaB ligand gene via protein kinase C and extracellular signal-regulated kinase pathways in MC3T3-E1 osteoblast-like cells. Archives Oral Biol. 52 (8), 778–785. 10.1016/j.archoralbio.2007.01.005 17306214

[B131] Oderda L. H. Young J. R. Asche C. V. Pepper G. A. (2012). Psychotropic-related hip fractures: Meta-analysis of first-generation and second-generation antidepressant and antipsychotic drugs. Ann. Pharmacother. 46 (7-8), 917–928. 10.1345/aph.1Q589 22811347

[B132] Oh-ie K. Miyazaki T. Koyama I. Hokari S. Komoda T. (2002). Altered bone turnover in chlorpromazine-challenged rats and its effect on 1alpha-hydroxyvitamin D3 administration *in vivo* . J. Bone Mineral Metabolism 20 (1), 21–27. 10.1007/s774-002-8442-7 11810412

[B133] Omérov M. Wistedt B. Bolvig-Hansen L. Larsen N. E. (1989). The relationship between perphenazine plasma levels and clinical response in acute schizophrenia. Prog. Neuropsychopharmacol. Biol. Psychiatry 13 (1-2), 159–166. 10.1016/0278-5846(89)90013-4 2664884

[B134] Papola D. Ostuzzi G. Thabane L. Guyatt G. Barbui C. (2018). Antipsychotic drug exposure and risk of fracture: A systematic review and meta-analysis of observational studies. Int. Clin. Psychopharmacol. 33 (4), 181–196. 10.1097/YIC.0000000000000221 29688914

[B135] Park K.-R. Kim E.-C. Hong J. T. Yun H.-M. (2018). Dysregulation of 5-hydroxytryptamine 6 receptor accelerates maturation of bone-resorbing osteoclasts and induces bone loss. Theranostics 8 (11), 3087–3098. 10.7150/thno.24426 29896304 PMC5996355

[B136] Paschou S. A. Mentzelopoulos P. Lambrinoudaki I. (2019). Antipsychotic therapies and bone health. Case Rep. Womens Health 25, e00160–e. 10.1016/j.crwh.2019.e00160 31867222 PMC6906724

[B137] Pasco J. A. Henry M. J. Sanders K. M. Kotowicz M. A. Seeman E. Nicholson G. C. (2004). Beta-adrenergic blockers reduce the risk of fracture partly by increasing bone mineral density: Geelong Osteoporosis Study. J. Bone Mineral Res. 19 (1), 19–24. 10.1359/JBMR.0301214 14753732

[B138] Patel K. R. Cherian J. Gohil K. Atkinson D. (2014). Schizophrenia: Overview and treatment options. P T 39 (9), 638–645. 25210417 PMC4159061

[B139] Perez D. M. (2020). α1-Adrenergic receptors in neurotransmission, synaptic plasticity, and cognition. Front. Pharmacol. 11, 581098. 10.3389/fphar.2020.581098 33117176 PMC7553051

[B140] Pillinger T. McCutcheon R. A. Vano L. Mizuno Y. Arumuham A. Hindley G. (2020). Comparative effects of 18 antipsychotics on metabolic function in patients with schizophrenia, predictors of metabolic dysregulation, and association with psychopathology: A systematic review and network meta-analysis. Lancet Psychiatry 7 (1), 64–77. 10.1016/S2215-0366(19)30416-X 31860457 PMC7029416

[B141] Pittenger M. F. Mackay A. M. Beck S. C. Jaiswal R. K. Douglas R. Mosca J. D. (1999). Multilineage potential of adult human mesenchymal stem cells. Science 284 (5411), 143–147. 10.1126/science.284.5411.143 10102814

[B142] Pożarowska K. Rusinek A. Rudziński G. Soroka E. Masiak J. (2021). New drugs in psychiatry - cariprazine, lurasidone, esketamine. Curr. Problems Psychiatry 22 (2), 111–124. 10.2478/cpp-2021-0010

[B143] Prieto E. Micó J. A. Meana J. J. Majadas S. (2010). Neurobiological bases of quetiapine antidepresant effect in the bipolar disorder. Actas Esp. Psiquiatr. 38 (1), 22–32. 20931407

[B144] Ramachandraiah C. T. Subramaniam N. Tancer M. (2009). The story of antipsychotics: Past and present. Indian J. Psychiatry 51 (4), 324–326. 10.4103/0019-5545.58304 20048463 PMC2802385

[B145] Ramaswamy G. Kim H. Zhang D. Lounev V. Wu J. Y. Choi Y. (2017). Gsα controls cortical bone quality by regulating osteoclast differentiation via cAMP/PKA and β-catenin pathways. Sci. Rep. 7 (1), 45140. 10.1038/srep45140 28338087 PMC5364530

[B146] Rampino A. Marakhovskaia A. Soares-Silva T. Torretta S. Veneziani F. Beaulieu J. M. (2019). Antipsychotic drug responsiveness and dopamine receptor signaling; old players and new prospects. Front. Psychiatry 9, 702. 10.3389/fpsyt.2018.00702 30687136 PMC6338030

[B147] Richtand N. Welge J. Logue A. Keck P. Strakowski S. McNamara R. (2007). Dopamine and serotonin receptor binding and antipsychotic efficacy. Neuropsychopharmacology 32:1715–1726. 10.1038/sj.npp.1301305 17251913

[B148] Rigler S. K. Shireman T. I. Cook-Wiens G. J. Ellerbeck E. F. Whittle J. C. Mehr D. R. (2013). Fracture risk in nursing home residents initiating antipsychotic medications. J. Am. Geriatr. Soc. 61 (5), 715–722. 10.1111/jgs.12216 23590366 PMC3656141

[B149] Rosenbloom A. L. (2010). Hyperprolactinemia with antipsychotic drugs in children and adolescents. Int. J. Pediatr. Endocrinol. 2010, 159402. 10.1155/2010/159402 20871665 PMC2943074

[B150] Sacchetti B. Funari A. Michienzi S. Di Cesare S. Piersanti S. Saggio I. (2007). Self-renewing osteoprogenitors in bone marrow sinusoids can organize a hematopoietic microenvironment. Cell 131 (2), 324–336. 10.1016/j.cell.2007.08.025 17956733

[B151] Saller C. F. Salama A. I. (1993). Seroquel: Biochemical profile of a potential atypical antipsychotic. Psychopharmacol. Berl. 112 (2-3), 285–292. 10.1007/BF02244923 7871032

[B152] Sato T. Arai M. Goto S. Togari A. (2010). Effects of propranolol on bone metabolism in spontaneously hypertensive rats. J. Pharmacol. Exp. Ther. 334 (1), 99–105. 10.1124/jpet.110.167643 20404011

[B153] Schreiber S. Pick C. G. (2021). The opioid interactions of the antipsychotic medications risperidone and amisulpride in mice and their potential use in the treatment of other non-psychotic medical conditions. Cell. Mol. Neurobiol. 41 (5), 1077–1084. 10.1007/s10571-020-01001-2 33184770 PMC11448585

[B154] Sekhar G. N. Fleckney A. L. Boyanova S. T. Rupawala H. Lo R. Wang H. (2019). Region-specific blood–brain barrier transporter changes leads to increased sensitivity to amisulpride in Alzheimer’s disease. Fluids Barriers CNS 16 (1), 38. 10.1186/s12987-019-0158-1 31842924 PMC6915870

[B155] Shapiro D. A. Renock S. Arrington E. Chiodo L. A. Liu L. X. Sibley D. R. (2003). Aripiprazole, a novel atypical antipsychotic drug with a unique and robust pharmacology. Neuropsychopharmacology 28 (8), 1400–1411. 10.1038/sj.npp.1300203 12784105

[B156] Shen S. P. Liu Y. Qiu H. Tsai K. Y. Wu H. C. Liang W. M. (2019). The risk of bone fracture after long-term risperidone exposure is not increased compared to other atypical antipsychotics: A retrospective cohort study. PLoS One 14 (9), e0221948. 10.1371/journal.pone.0221948 31487309 PMC6728018

[B157] Shirley M. Perry C. M. (2014). Aripiprazole (abilify MAINTENA®): A review of its use as maintenance treatment for adult patients with schizophrenia. Drugs 74, 1097–1110. 10.1007/s40265-014-0231-7 24969315

[B158] Siafis S. Tzachanis D. Samara M. Papazisis G. (2018). Antipsychotic drugs: From receptor-binding profiles to metabolic side effects. Curr. Neuropharmacol. 16 (8), 1210–1223. 10.2174/1570159X15666170630163616 28676017 PMC6187748

[B159] Siddappa R. Mulder W. Steeghs I. van de Klundert C. Fernandes H. Liu J. (2009). cAMP/PKA signaling inhibits osteogenic differentiation and bone formation in rodent models. Tissue Eng. Part A 15 (8), 2135–2143. 10.1089/ten.tea.2008.0512 19231969

[B160] Stahl S. M. (2018). Beyond the dopamine hypothesis of schizophrenia to three neural networks of psychosis: Dopamine, serotonin, and glutamate. CNS Spectrums 23 (3), 187–191. 10.1017/S1092852918001013 29954475

[B161] Strange P. G. (2008). Antipsychotic drug action: Antagonism, inverse agonism or partial agonism. Trends Pharmacol. Sci. 29 (6), 314–321. 10.1016/j.tips.2008.03.009 18471899

[B162] Stubbs B. Gaughran F. Mitchell A. J. De Hert M. Farmer R. Soundy A. (2015). Schizophrenia and the risk of fractures: A systematic review and comparative meta-analysis. General Hosp. Psychiatry 37 (2), 126–133. 10.1016/j.genhosppsych.2015.01.004 25666994

[B163] Suga S. Goto S. Togari A. (2010). Demonstration of direct neurite–osteoclastic cell communication *in vitro* via the adrenergic receptor. J. Pharmacol. Sci. 112 (2), 184–191. 10.1254/jphs.09283fp 20093791

[B164] Takeda S. Elefteriou F. Levasseur R. Liu X. Zhao L. Parker K. L. (2002). Leptin regulates bone formation via the sympathetic nervous system. Cell 111 (3), 305–317. 10.1016/s0092-8674(02)01049-8 12419242

[B165] Togari A. (2002). Adrenergic regulation of bone metabolism: Possible involvement of sympathetic innervation of osteoblastic and osteoclastic cells. Microsc. Res. Tech. 58 (2), 77–84. 10.1002/jemt.10121 12203706

[B166] Togari A. Arai M. Mizutani S. Mizutani S. Koshihara Y. Nagatsu T. (1997). Expression of mRNAs for neuropeptide receptors and β-adrenergic receptors in human osteoblasts and human osteogenic sarcoma cells. Neurosci. Lett. 233 (2), 125–128. 10.1016/s0304-3940(97)00649-6 9350848

[B167] Tollefson G. D. Taylor C. C. (2000). Olanzapine: Preclinical and clinical profiles of a novel antipsychotic agent. CNS Drug Rev. 6 (4), 303–363. 10.1111/j.1527-3458.2000.tb00155.x

[B168] Tsai K.-Y. Lee C.-C. Chou Y.-M. Shen S.-P. Su C.-Y. Wu H.-C. (2014). The risks of major osteoporotic fractures in patients with schizophrenia: A population-based 10-year follow-up study. Schizophrenia Res. 159 (2), 322–328. 10.1016/j.schres.2014.09.032 25445622

[B169] Tseng P. T. Chen Y. W. Yeh P. Y. Tu K. Y. Cheng Y. S. Wu C. K. (2015). Bone mineral density in schizophrenia: An update of current meta-analysis and literature review under guideline of PRISMA. Med. Baltim. 94 (47), e1967. 10.1097/MD.0000000000001967 PMC5058960 26632691

[B170] van Gastel J. Hendrickx J. O. Leysen H. Santos-Otte P. Luttrell L. M. Martin B. (2018). β-Arrestin based receptor signaling paradigms: Potential therapeutic targets for complex age-related disorders. Front. Pharmacol. 9, 1369. 10.3389/fphar.2018.01369 30546309 PMC6280185

[B171] Van Putten T. Marder S. R. Wirshing W. C. Aravagiri M. Chabert N. (1991). Neuroleptic plasma levels. Schizophr. Bull. 17 (2), 197–216. 10.1093/schbul/17.2.197 1679251

[B172] Vasiliu O. (2022). Third-generation antipsychotics in patients with schizophrenia and non-responsivity or intolerance to clozapine regimen: What is the evidence? Front. Psychiatry 13, 1069432. 10.3389/fpsyt.2022.1069432 36523870 PMC9744942

[B173] Walker E. C. McGregor N. E. Poulton I. J. Solano M. Pompolo S. Fernandes T. J. (2010). Oncostatin M promotes bone formation independently of resorption when signaling through leukemia inhibitory factor receptor in mice. J. Clin. Invest. 120 (2), 582–592. 10.1172/JCI40568 20051625 PMC2810087

[B174] Wang C.-X. Ge X.-Y. Wang M.-Y. Ma T. Zhang Y. Lin Y. (2020). Dopamine D1 receptor-mediated activation of the ERK signaling pathway is involved in the osteogenic differentiation of bone mesenchymal stem cells. Stem Cell Res. Ther. 11 (1), 12. 10.1186/s13287-019-1529-x 31900224 PMC6942280

[B175] Wang H. Xu J. Lazarovici P. Quirion R. Zheng W. (2018). cAMP response element-binding protein (CREB): A possible signaling molecule link in the pathophysiology of schizophrenia. Front. Mol. Neurosci. 11, 255. 10.3389/fnmol.2018.00255 30214393 PMC6125665

[B176] Wang J. Jiang F. Yang Y. Zhang Y. Liu Z. Qin X. (2021). Description of three new species of the leafhopper genus idioscopus baker, (Hemiptera: Cicadellidae: Eurymelinae) from yunnan, China. BMC Psychiatry 21 (1), 375–381. 10.11646/zootaxa.4995.2.10 34810564

[B177] Wang L. Han L. Xue P. Hu X. Wong S.-W. Deng M. (2021). Dopamine suppresses osteoclast differentiation via cAMP/PKA/CREB pathway. Cell Signal 78, 109847. 10.1016/j.cellsig.2020.109847 33242564 PMC8691485

[B178] Watanabe Y. Yamada S. Otsubo T. Kikuchi T. (2020). Brexpiprazole for the treatment of schizophrenia in adults: An overview of its clinical efficacy and safety and a psychiatrist’s perspective. Drug Des. Devel Ther. 14, 5559–5574. 10.2147/DDDT.S240859 PMC7755340 33376301

[B179] Watson D. J. G. Loiseau F. Ingallinesi M. Millan M. J. Marsden C. A. Fone K. C. F. (2012). Selective blockade of dopamine D3 receptors enhances while D2 receptor antagonism impairs social novelty discrimination and novel object recognition in rats: A key role for the prefrontal cortex. Neuropsychopharmacology 37 (3), 770–786. 10.1038/npp.2011.254 22030711 PMC3261029

[B180] Weinstein J. J. Chohan M. O. Slifstein M. Kegeles L. S. Moore H. Abi-Dargham A. (2017). Pathway-specific dopamine abnormalities in schizophrenia. Biol. Psychiatry 81 (1), 31–42. 10.1016/j.biopsych.2016.03.2104 27206569 PMC5177794

[B182] Westbroek I. van der Plas A. de Rooij K. E. Klein-Nulend J. Nijweide P. J. (2001). Expression of serotonin receptors in bone. J. Biol. Chem. 276 (31), 28961–28968. 10.1074/jbc.M101824200 11387323

[B183] Winship I. R. Dursun S. M. Baker G. B. Balista P. A. Kandratavicius L. Maia-de-Oliveira J. P. (2019). An overview of animal models related to schizophrenia. Can. J. Psychiatry 64 (1), 5–17. 10.1177/0706743718773728 29742910 PMC6364139

[B184] Wysokiński A. Kozłowska E. Szczepocka E. Łucka A. Agier J. Brzezińska-Błaszczyk E. (2021). Expression of dopamine D1−4 and serotonin 5-ht1a-3A receptors in blood mononuclear cells in schizophrenia. Front. Psychiatry 12, 645081. 10.3389/fpsyt.2021.645081 33776821 PMC7988204

[B185] Yadav V. K. Ryu J. H. Suda N. Tanaka K. F. Gingrich J. A. Schütz G. (2008). Lrp5 controls bone formation by inhibiting serotonin synthesis in the duodenum. Cell 135 (5), 825–837. 10.1016/j.cell.2008.09.059 19041748 PMC2614332

[B186] Yang A. C. Tsai S.-J. (2017). New targets for schizophrenia treatment beyond the dopamine hypothesis. Int. J. Mol. Sci. 18 (8), 1689. 10.3390/ijms18081689 28771182 PMC5578079

[B187] Yang H. Xu Y. Zhu M. Gu Y. Zhang W. Shao H. (2016). Inhibition of titanium-particle-induced inflammatory osteolysis after local administration of dopamine and suppression of osteoclastogenesis via D2-like receptor signaling pathway. Biomaterials 80, 1–10. 10.1016/j.biomaterials.2015.11.046 26695376

[B188] Yun H. M. Park K. R. Hong J. T. Kim E. C. (2016). Peripheral serotonin-mediated system suppresses bone development and regeneration via serotonin 6 G-protein-coupled receptor. Sci. Rep. 6, 30985. 10.1038/srep30985 27581523 PMC5007490

[B189] Zhang L. Shi G. (2016). Gq-coupled receptors in autoimmunity. J. Immunol. Res. 2016, 3969023. 10.1155/2016/3969023 26885533 PMC4739231

[B190] Zhang R. Edwards J. R. Ko S. Y. Dong S. Liu H. Oyajobi B. O. (2011). Transcriptional regulation of BMP2 expression by the PTH-CREB signaling pathway in osteoblasts. PLoS One 6 (6), e20780. 10.1371/journal.pone.0020780 21695256 PMC3111437

[B191] Zhao J. Deng Y. Jiang Z. Qing H. G. (2016). G protein-coupled receptors (GPCRs) in alzheimer's disease: A focus on BACE1 related GPCRs. Front. Aging Neurosci. 8, 58. 10.3389/fnagi.2016.00058 27047374 PMC4805599

[B192] Zhong X. P. Xia W. F. (2021). Regulation of bone metabolism mediated by β-adrenergic receptor and its clinical application. World J. Clin. Cases 9 (30), 8967–8973. 10.12998/wjcc.v9.i30.8967 34786380 PMC8567525

[B193] Zhu J. Feng C. Zhang W. Wang Z. Zhong M. Tang W. (2022). Activation of dopamine receptor D1 promotes osteogenic differentiation and reduces glucocorticoid-induced bone loss by upregulating the ERK1/2 signaling pathway. Mol. Med. 28 (1), 23. 10.1186/s10020-022-00453-0 35189819 PMC8862482

[B194] Zhu X. Zheng H. (2021). Factors influencing peak bone mass gain. Front. Med. 15 (1), 53–69. 10.1007/s11684-020-0748-y 32519297

